# Nursing Informatics and Undergraduate Nursing Curricula: A Scoping Review

**DOI:** 10.3390/nursrep16020042

**Published:** 2026-01-27

**Authors:** Lisa Reid, Didy Button, Katrina Breaden, Mark Brommeyer

**Affiliations:** 1 College of Nursing and Health Sciences, Flinders University, Adelaide, SA 5042, Australia; didy.button@flinders.edu.au (D.B.); katrina.breaden@flinders.edu.au (K.B.); 2 College of Business, Government and Law, Flinders University, Adelaide, SA 5042, Australia; mark.brommeyer@flinders.edu.au

**Keywords:** nursing informatics, nursing education, digital health, workforce readiness

## Abstract

**Introduction**: Nursing informatics aims to improve patient care through rapid access to patient data, systematic assessment, a reduction in clinical errors, evidence-based practice, cost-effectiveness, and improved patient outcomes and safety. **Background:** Despite being the largest workforce in healthcare, nurses are not being adequately prepared to use nursing informatics, and this has been attributed to poor digital literacy, limited professional development, and a lack of undergraduate informatics education. **Objectives**: This scoping review aims to review contemporary published literature on the benefits, barriers, and enablers for embedding nursing informatics into undergraduate nursing education with a focus on the Australian healthcare context. **Methods**: A scoping review was conducted using the PRISMA-ScR checklist and the JBI Manual for evidence synthesis in adherence with an a priori scoping review protocol. A comprehensive search of JBI, Cochrane, CINAHL, Ovid, ProQuest, PubMed, and Scopus databases was performed. Two reviewers independently screened the results via Covidence, with discrepancies resolved via a third reviewer. **Results**: Two searches were conducted for this scoping review. In the first search, a total of 3227 articles were identified through database searches, with an additional 76 articles identified through bibliographic and grey literature searches. Following duplicate removal and screening, 46 articles met the inclusion criteria. In the second search, a total of 1555 articles were identified, and after duplicate removal and screening, 16 articles met the inclusion criteria. Duplicate removal during the second search round included those articles identified in the first search. The combined searches resulted in a total of 62 sources for this review. **Conclusions**: Despite the early adoption of nursing informatics in Australia in the 1980s, barriers remain to effective nursing informatics engagement and proficiency, including a lack of understanding of nursing informatics, limited infrastructure and resources, inadequate digital literacy of students and faculty, and the evolving nature of nursing informatics. Definitions of nursing informatics and associated fields, development of university faculty competency, access to digital health technologies, competency standards, digital literacy of the student cohort, faculty digital proficiency, and leadership from professional nursing bodies are all viewed as integral foundations for the development of student competency in nursing informatics.

## 1. Introduction

Healthcare is underpinned by the desire to provide safe, cost-effective, efficient, and accessible care that prioritises patient outcomes and uses the latest evidence to inform practice. Over the past 70 years, healthcare has increasingly used information and communication technologies to improve the safety and efficacy of healthcare [1], develop evidence-based practices [2], provide data to leverage change and inform healthcare policy [3], and articulate the role of healthcare in society [4]. Reflecting the digital revolution in healthcare, the health informatics fields have sought to integrate health information and knowledge with information and communication technologies to promote optimal patient health outcomes [5,6]. The advancement of digital technologies has transformed and changed the way in which healthcare is delivered [7,8,9], with nursing informatics linking technology with nursing practice and aiming to provide more cost-effective, efficient, accessible, and safer care [10]. The ability to live, work, participate and thrive in a digital world is imperative for nurses [11] with nursing informatics informing clinical decision-making and enhancing patient outcomes [12,13].

Computers were first introduced to nursing care in the 1950s [1,14,15], with the first global conference on medical informatics held in 1974 in Stockholm, Sweden and five papers presented on nursing informatics at this time [15,16]. The term nursing informatics was first proposed by Scholes and Barber in 1976 [17] and arose “from the French term, informatique” referring to the use of computers [15] (p. 177). Globally, efforts to promote nursing informatics included the formation of the International Medical Informatics Association–Nursing Informatics working group in 1982 [18,19], the Canadian Nursing Informatics Association in 2002 [20], the Alliance for Nursing Informatics in 2004 [21], and the Technology Informatics Guiding Education Reform (TIGER) Initiative [22]. Despite these efforts, a lack of understanding of nursing informatics has persisted [23,24], with “at least 14 definitions of nursing informatics” emerging over the past four decades [25] (p. 1034), confusing the nature of nursing informatics and its relevance to nursing practice [10]. Further compounding this issue has been a lack of consensus on health informatics and digital health terminologies [26,27,28], with a lack of consistent nursing informatics competencies worldwide [29,30]. Disparate undergraduate nursing education regarding nursing informatics [30] and a lack of university faculty with nursing informatics competence and expertise [31] have resulted in a healthcare workforce not adequately prepared to work within the digital health sphere [32]. In response to some of these issues, a recent integrative review [33] of the current nursing and midwifery contribution to leading digital health policy has called for digital health skills, competencies, and capabilities to be integrated into health professional education.

Within the Australian healthcare context, the integration of nursing informatics into undergraduate nursing curricula has also remained fragmented. In Australia, nursing informatics first emerged around 1984, with Hovenga [34] noting that nursing informatics has “played a very significant role in creating an awareness about the discipline and in educating nurses and other health professionals regarding the use of digitised health information using the technologies available”. In 1991, Nursing Informatics Australia (NIA) was formed, following the International Medical Informatics Association—Nursing Informatics Conference in Melbourne, Australia, to bring together nursing informatics groups from across Australia [35]. In response to the changing nature of healthcare, the Australia Ministers’ Advisory Council was established in 2003 to develop a national digital action plan. In 2008, the National E-Health Transition Authority (NEHTA) published the National E-Health Strategy, a guide for the ongoing development of eHealth in Australia [36]. In 2016, the Australian Digital Health Agency (ADHA) was established by the Australian Federal Government as a statutory authority responsible for the national digital health strategy [37]. In 2017, ADHA released safe, seamless, and secure evolving healthcare to meet the needs of modern Australia—Australia’s National Digital Health Strategy [38] with Statement six stating that by 2022, a collaboration between the agency and other key parties would develop digital health resources to support the development of a digitally literate healthcare workforce. Subsequently, in 2020, the National Nursing and Midwifery Digital Health Capability Framework was released by ADHA [39] (p. 8), with the intention of outlining “the capabilities required to support individuals and organisations in extending their digital health development rather than providing a rigid set of competencies”. However, despite these efforts, contemporary Australian literature has highlighted a lack of consistent nursing informatics education across Australian undergraduate nursing programs [10,40,41,42,43]. In response to these identified deficits, this scoping review maps contemporary evidence through the themes of enablers, barriers, and benefits of nursing informatics integration into undergraduate nursing curricula and translates this evidence into recommendations for the Australian healthcare system.

## 2. Materials and Methods

Scoping reviews can be undertaken with the objective of providing an overview of existing evidence, mapping key concepts, defining working definitions, and providing a broad overview of a topic [44]. In A scoping review of scoping reviews, Pham et al. [45] found that the most common reasons for conducting a scoping review were for exploring the breadth, extent and range of literature, mapping and summarising the evidence, and informing future research. The purpose of this scoping review is to explore contemporary published literature on embedding nursing informatics into undergraduate nursing education.

In 2005, Arksey and O’Malley [46] published the seminal paper: Scoping studies: towards a methodological framework, with a five-step methodological framework for scoping reviews to enhance transparency, rigour, and replicability. In 2010, Levac et al. [47], in Scoping studies: advancing the methodology, provided further recommendations to strengthen Arksey and O’Malley’s scoping study framework. In 2013, the JBI (Joanna Briggs Institute) Scoping Review Methodology Group was formed to develop a comprehensive guide for the conduct of scoping reviews, which built on the seminal work of Arksey and O’Malley [46] and Levac et al. [47] and in collaboration with other experts in scoping reviews, published the Preferred Reporting Items for Systematic Reviews (PRISMA) Statement for Scoping Reviews—PRISMA-ScR [48]. The JBI Manual for Evidence Synthesis: 2024 Edition [44] incorporates the Prisma-ScR into the JBI scoping review methodology, proposes enhancements to the Arksey and O’Malley framework, and has nine steps [44,49]:

1. Defining and aligning the objective/s and questions/s
2. Developing and aligning the inclusion question/s
3. Describing the planned approach to evidence searching, selection, data extraction, and presentation of the evidence
4. Searching for the evidence
5. Selecting the evidence
6. Extracting the evidence
7. Analysis of the evidence
8. Presentation of the results
9. Summarising the evidence in relation to the purpose of the review, making conclusions and noting any implications of the findings

The JBI Manual for Evidence Synthesis: 2024 Edition [44] requires the development of an a priori protocol before the commencement of the scoping review, stating “as with all well-conducted systematic reviews, an a priori protocol must be developed before undertaking the scoping review”. A scoping review was conducted using the PRISMA-ScR Checklist [48] and JBI Manual for Evidence Synthesis [50], which are guided by the seminal work of Arksey and O’Malley [46], Davis et al. [51], Levac et al. [47], and Peters et al. [50]. The completed PRISMA-ScR checklist is provided as a Supplementary File.

### 2.1. Protocol and Registration

An a priori protocol for this scoping review was registered on the Open Science Framework (OSF) Platform on 10 August 2022 (https://osf.io/7qe39/overview). The scoping review protocol was subsequently published [52]. Please note: this scoping review reflects a section of the original review.

### 2.2. Review Question

Step 1 of the JBI Scoping Review Framework [44] is defining and aligning the objective/s and questions/s. This scoping review aimed to address whether a distinct body of knowledge on nursing informatics could be further developed to inform undergraduate nursing curricula development within the Australian tertiary system by including a review of benefits, barriers, and enablers. The scoping review questions were:

- What are the benefits of nursing informatics?
- What are the barriers to the integration of nursing informatics in undergraduate nursing curricula?
- What are the enablers to the integration of nursing informatics in undergraduate nursing curricula?

### 2.3. Eligibility Criteria

Step 2 of the JBI Scoping Review Framework [44] is developing and aligning the inclusion questions. Peters et al. [53] recommend the use of the PCC mnemonic (population, concept and context) to identify the focus and context of the review. The population of this scoping review was undergraduate nursing students, the concept was nursing informatics, and the context was education. To be included in this scoping review, papers need to include nursing informatics education for undergraduate nursing students at any time during a Bachelor of Nursing program (or equivalent). For this scoping review, nursing informatics is defined as “the specialty that integrates nursing science with multiple information and analytical sciences to identify, define, manage, and communicate data, information, knowledge, and wisdom in nursing practice”, as defined in the seminal work of Staggers and Thompson [54] (p. 260) and cited in the Nursing Informatics Position Statement, published in 2017 by the Australian College of Nursing, Health Informatics Society of Australia and Nursing Informatics Australia [55].

Sources of information were included if they were published between 2015 and 2022 and described curriculum recommendations (including barriers to implementing nursing informatics education). The subsequent desktop review included articles from 2022 to 2025 (justification for this review is addressed in Section 2.6). Articles published in languages other than English and outside of the timeline parameters were also excluded. It is noted that the exclusion of non-English sources was a limitation of this approach, as seminal sources published in other languages may have been omitted. However, this decision reflected the focus on the Australian healthcare context.

### 2.4. Information Sources and Search Strategy

Step 3 of the JBI Scoping Review Framework [44] is describing the planned approach to evidence searching, selection, data extraction, and presentation of the evidence. A scoping review search strategy should be as comprehensive as possible within time constraints and seek to identify both published and unpublished literature with a three-step strategy [44]. The planned approach was described in the a priori protocol both in the Open Science Framework (OSF) Platform (https://osf.io/7qe39/overview) and the published protocol [52].

Step 4 of the JBI Scoping Review Framework [44] is searching for the evidence. A search of both Cochrane and the JBI databases was performed in December 2020, and no existing review protocols were identified. CINAHL, Ovid, ProQuest, PubMed, and Scopus were searched for articles that met the inclusion criteria, including scholarly journals, books, reports, conference papers and proceedings. A subsequent search of the grey literature and bibliography sources was performed following the review of databases. The first step required an initial limited search of two databases and an analysis of text words in the title and abstract. In the second step, all identified keywords were used across all included databases. The final step required that the reference lists of selected full-text sources should be examined and included in the review (if relevant to the phenomenon of interest). The final search string was “nursing informatics” AND 2015–2022 AND (educat* OR curric* OR pedagog* OR “nursing education) AND (profic* OR engage* OR accept* OR capabil* OR competenc* OR knowledg* OR “core content” OR standards OR practice). To be generalisable to the Australian healthcare system for this scoping review article, it was decided to solely include articles from those countries who have “comparable regulatory approaches, standards for education and registration, processes and procedures for the registration” of nurses [56]; these comparable jurisdiction include: United Kingdom, Ireland, United States, Canadian provinces of British Columbia and Ontario, Singapore, Spain, and New Zealand, under the *Trans-Tasman Mutual Recognition Act 1997* (Cth) [57]. The subsequent desktop review search criteria are addressed in Section 2.6.

### 2.5. Data Extraction and Analysis

Step 5 of the JBI Scoping Review Framework [44] is selecting the evidence. Following searches of the database, using the a priori protocol [52] and the removal of duplicate articles, selection of sources of information was performed independently by four reviewers (L.R., D.B., K.B., and M.B.) using the Covidence software platform (June 2022 version). Covidence is “a web-based collaboration software platform that streamlines the production of systematic and other literature reviews” and aids in the uploading of search results, the screening of abstracts and full text, completing data collection, review by two or more reviewers and exporting of data [58]. First Pass—Title and Abstract Screening required a reviewer (L.R.) to screen each article in consultation with the other reviewers (D.B., K.B., and M.B.). Double-screening of 10 articles was performed before First Pass—Title and Abstract Screening to enhance internal consistency. Please refer to Table 1 for exclusion criteria.

To enhance the trustworthiness of the screening process, an Excel spreadsheet was developed, and each excluded source was categorised. Second Pass—Full-Text Screening involved examining the full text of each article to determine if it met the inclusion criteria and providing coherent reasons for exclusion of articles. During the second screening process, two reviewers read the full text of articles, and these articles were discussed in weekly meetings. Critical appraisal of the sources of evidence is an optional item within the PRISMA Extension for Scoping Reviews (PRISMA-ScR): Checklist and Explanation [48], and “included sources of evidence are typically not critically appraised for scoping reviews”. Therefore, for this review, a critical appraisal was not performed.

Step 6 of the JBI Scoping Review Framework [44] is extracting the evidence. Extraction of data performed independently by four reviewers (L.R., D.B., K.B., and M.B.) using the Covidence software platform. During data extraction, two reviewers read the full text of articles, and Covidence was used to extract data; discrepancies in data extraction were addressed in weekly meetings. Extracted data included authors, year of publication, aim/purpose of study, enablers, barriers, benefits, and use of competency frameworks.

Step 7 of the JBI Scoping Review Framework [44] is analysis of the evidence. Methods of data analysis in a scoping review may include descriptive qualitative content analysis, frequency counts of the population, concepts and context (PCC) or basic coding [44]. “The most important consideration regarding analysis is that the authors are transparent and explicit in the approach they have taken, including justifying their approach and clearly reporting any analyses, and as much as possible planned and stipulated a priori” [44] (n.p.). A decision was made to use a quantitative approach, identifying absolute frequency counts of population, concepts and context and a qualitative content analysis framework with manual coding to describe the key information relevant to the scoping review questions.

Frequency counts were calculated using the Covidence data extraction template data for the PCC mnemonic. Qualitative content analysis [59] was used to elicit meaning from the data by using the stages of decontextualisation, recontextualisation, categorisation, and compilations to code and categorise data. Qualitative content analysis requires the in-depth reading of text, reorganisation of relevant sections into categories, and then creating interpretations or narratives [60], with manifest content codes in the initial stages of analysis and latent content emerging from the researchers’ interpretation of the text [61]. This scoping review used an abductive inference approach, which moves between inductive and abductive approaches, bridging “the gap between descriptive accounts of texts and what they mean, refer to, entail, provoke, or cause” [60]. A manual code book was maintained, including the use of an Excel spreadsheet, with weekly meetings between all researchers (L.R., D.B., K.B., and M.B.) to address coding and establish intercoder agreement. Disagreements regarding coding between two researchers were addressed by group consensus.

### 2.6. Subsequent Desktop Review

An updated search can be conducted if new sources of information may result in changes to study findings [62]. An update of the initial scoping review was conducted in November 2025 by means of a desktop review to identify new and relevant sources of information, thereby reflecting the rapidly evolving nature of nursing informatics. “Desktop research involves the use of existing sources of information to gather data and insights on a topic of interest and is often used to support primary research” [62]. The purpose of the desktop review was to include additional contemporary sources of information that were outside of the initial scoping review timeline inclusion criteria, which may result in a change in findings. This updated search used the same search strategy as the original scoping review, but the search was limited to sources from 2022 to 2025 in the Scopus database, and the grey literature was not searched. Non-inclusion of the grey literature is addressed in Section Limitations. The inclusion criteria included articles published in English from 2022 to 2025; publications that were peer-reviewed, empirical and were relevant to the Australian healthcare context. Three reviewers (L.R., K.B., and M.B.) reviewed the sources of information, and the combined searches resulted in 62 sources included in this review.

The sources of information from both the initial scoping review and the subsequent desktop review were combined in Covidence. Duplicates were removed, and the First Pass—Title and Abstract Screening required a reviewer (L.R.) to screen each article in consultation with the other reviewers (K.B. and M.B.). Second Pass—Full-Text Screening of both reviews was examined to determine if they met the inclusion criteria, with coherent reasons for exclusion of articles documented. Refer to Table 1 for exclusion criteria. Critical appraisal of the sources of evidence was not performed (as previously detailed).

## 3. Results

Step 8 of the JBI Scoping Review Framework [44] is presenting the results. In the presentation of results, Peters et al. [53] suggest the use of two sections: the first section describing the search strategy results (with the inclusion of the PRISMA flow diagram) and the second section detailing the key information relevant to the scoping review questions. Providing the results in this format will allow reviewers to identify gaps in the literature and appropriately map the existing evidence. Step 9 of the JBI Scoping Review Framework [44] is summarising the evidence in relation to the purpose of the review, making conclusions and noting any implications of the findings—this step is addressed in the discussion section.

A total of 3227 articles were identified in the original scoping review through the database searches, with the grey literature (*n* = 13) and bibliography searches (*n* = 63) resulting in 76 additional articles. Following duplicate removal (*n* = 833), First Pass—Title and Abstract Screening (*n* = 2470) excluded 2356 articles. Second Pass—Full-Text Screening resulted in the exclusion of a further 68 articles (see PRISMA Diagram). A total of 46 articles met the inclusion criteria for the final scoping review.

**Figure 1:**
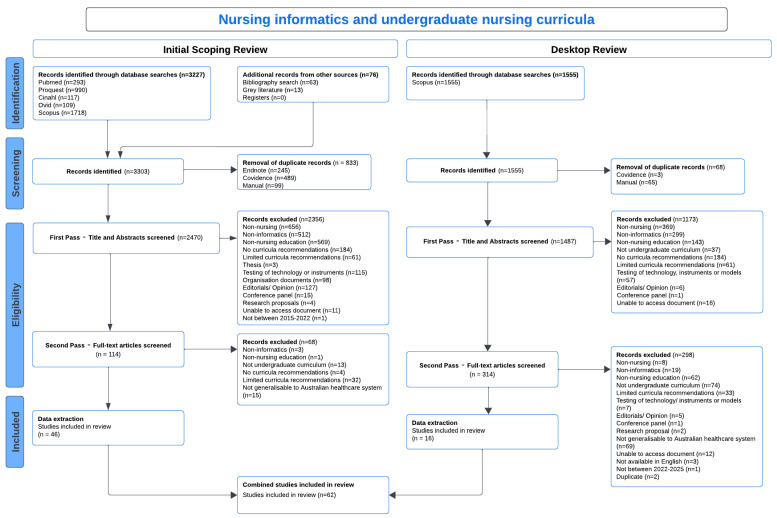
PRISMA diagram.

The subsequent desktop review identified a total of 1555 articles from the Scopus database. These articles were compared with the original scoping review to ensure that relevant sources of information from 2015 to 2025 were included, with duplicates removed. Following duplicate removal (*n* = 68), First Pass—Title and Abstract Screening (*n* = 1487) excluded 1173 articles. Second Pass—Full-Text Screening (*n* = 314) resulted in the exclusion of a further 298 articles (see Figure 1. PRISMA Diagram). A total of 16 articles met the inclusion criteria for the additional desktop review and were extracted using a data extraction template on Covidence. The information, relating to the scoping review, from these sources was integrated into the findings section. As per previous statements, sources of evidence for both the original scoping and the subsequent reviews were included for this article if the setting aligned with the Australian healthcare context. The data extraction table is provided as a Supplementary File.

### 3.1. Characteristics of Sources of Evidence

The selected studies came from multiple sites, including Australia [24,41,63,64,65,66,67,68,69,70,71,72,73,74,75,76,77,78], Canada [79,80,81,82,83,84,85,86,87,88,89,90,91], New Zealand [92], the United Kingdom [23,93,94], the United States of America [95,96,97,98,99,100,101,102,103,104,105,106,107,108,109,110,111,112,113,114,115,116,117], and across a range of sites [22,118,119].

### 3.2. Benefits

Luo and Kalman [112] (p. 21) noted that “Nurses’ technological knowledge of, skills in, and attitudes toward new technologies in the healthcare setting are critical to improving healthcare outcomes”.

#### 3.2.1. Benefits of Nursing Informatics in the Clinical Setting

The benefits of nursing informatics in the clinical setting were addressed in the selected studies, which include rapid and systematic patient assessment [24,65,93,101,103,105,106,109] supported by improved [24,41,63,64,65,66,67,68,69,70,71,72,73,74,75,76,77,78] data management [24,65,69,74,75,76,77,78,85,86,87,88,93,94,98,99,100,101,103,104,105,107,108,110,111,112,114,116,117,120,121], clinical reminders [101], improved interdisciplinary communication [22,65,73,87,93,97,110,112,119,120], and a reduction in clinical errors [79,95,115]. Further benefits include improved time management [69,98], cost-effectiveness and improved resource management [24,65,67,93,101,109,115,118], evidence-based practice and decision-support [22,24,67,68,71,73,74,85,87,89,90,91,93,97,99,100,101,104,105,107,108,112,116,119]. Patient self-monitoring and communication [23,65,72,74,87,97,102,109], population healthcare [11,93,101], and accessible care [84,86,88,102,104,109,114] were other notable benefits of nursing informatics. As identified by Foster and Sethares [107] (p. 1), “The current expanded use of computers and information systems in healthcare means that all healthcare workers, especially nurses will need to interface with multiple technological sources to either enter or extract data to aid them in caring for patients”. Therefore, nursing informatics education is integral to contemporary healthcare [22,23,24,41,63,64,65,67,68,69,71,72,73,74,75,76,77,78,79,80,81,82,84,85,86,87,88,89,90,91,92,93,94,95,96,97,98,99,100,101,102,103,104,105,107,108,109,110,111,112,114,115,116,117,118,119,121].

#### 3.2.2. Benefits of Nursing Informatics in Undergraduate Nursing Education

The benefits of nursing informatics in undergraduate nursing education include recognition of the importance of nursing informatics [106,115,117,118], development of digital literacy [23,41,67,71,72,74,75,76,77,78,81,82,83,84,86,87,88,89,90,97,98,99,103,104,106,113,121,122], the development of critical thinking and clinical reasoning [67,75,86,97,109,112,115,117], integrating theory and practice [64,83,99], understanding the importance of data [77,83,86,88,104,112,115,121], how to use data ethically [65,67,69,83,84,86,88,97,99,101,103,107,110,118], and the ability access evidence-based materials [71,81]. Finally, practice using electronic health records/electronic medical records, barcode medication administration/electronic medication administration records, telehealth, health informatics laboratories, and clinical support tools [24,41,63,75,76,78,79,80,84,86,95,96,97,99,100,101,103,106,109,110,112,113,114,115,117,121] was viewed as genuine and authentic real-world preparation [24,79,80,83,88,96,103,114] that enhanced the workplace readiness of graduates [63,64,65,74,86,105,109,110,112]. These benefits were highlighted by Harerimana et al. [67] (p. 527), when stating that “Embedding nursing informatics into the undergraduate nursing curriculum enhances nursing students’ digital health literacy, whilst preparing them to use health information systems and technological innovations to support their learning both at university and in the clinical environment”.

### 3.3. Barriers

Barriers to nursing informatics education emerged as a theme from the selected sources of evidence and were defined (by the authors) as anything preventing the effective integration of nursing informatics into undergraduate nursing education and associated clinical practice. This theme was underpinned by the sub-themes of understanding of nursing informatics, infrastructure and resources, student access to digital technologies, digital literacy of students and faculty, the evolving nature of nursing informatics, faculty responses to nursing informatics, and nursing informatics competencies and resources.

#### 3.3.1. Understanding of Nursing Informatics

The selected sources of evidence highlighted a lack of understanding of nursing and health informatics [23,24,66,78,85,98,116,118,122], limited understanding of nursing informatics applications, including electronic health records and handheld devices [22,24,78,80,102,106], and a lack of consistent taxonomy and language related to nursing informatics [87,119], as barriers to the effective integration of nursing informatics into undergraduate nursing programs. Chauvette et al. [81] (p. 7) noted that “faculty perceived level of NIC [nursing informatics competency] was largely based on their ability to work with digital tools to support pedagogical activities”, whilst Kleib and Olson [85] identified that faculty may attribute online learning skills to the nursing informatics competence. This confusion was still evident in the subsequent review [82,98,104], and reflects an ongoing issue identified by Graves and Corcoran [123] (p. 228), in 1989, who stated “nursing informatics is considered an integral part of the science of nursing and not simply a branch of computer science or information science applied to nursing”.

#### 3.3.2. Infrastructure and Resources

Significant barriers facing faculty, tertiary institutions, nurses, and undergraduate nursing students, when accessing nursing informatics education and resources, were identified. Computer crashes, power outages, hardware and software not working [71,76,80,87,95,102,119], a lack of technical support and poor infrastructure [24,41,67,92,100,106,108,109,118,119,121], poor internet connectivity and speed [71,87,96,108], and the development, and cost and maintenance of hardware and software [24,30,41,68,78,92,97,100,106,108,109,117,118,119,121] were all identified as barriers to the effective integration of nursing informatics into undergraduate nursing programs and associated clinical practice. Whilst not specifically addressed by the sources of information, the digital divide, defined as the gap between those who have access to digital technologies and those who do not [124], is an inherent issue in Australia, with almost a quarter of all Australians missing out on “the benefits that online connectivity provides” [125].

#### 3.3.3. Student Access to Digital Technologies

The selected sources also identified the challenges associated with a lack of student access to digital technologies and associated resources. A lack of access to digital health technologies due to access/supply, governance/policy, and privacy/ethics was identified in the sources of evidence. A lack of access to devices and associated software [41,67,81,85,92,97,100,109,110,126] was further complicated by university and clinical setting policies on student use of mobile devices [71,81] and digital technologies, including electronic health records [66,67,92,96,97,112,121]. Concerns regarding the legal and ethical implications of undergraduate nursing students accessing patient information were viewed as further inhibitory factors [67,71,97].

#### 3.3.4. Digital Literacy of Students and Faculty

Prensky [127,128] coined the term digital native, to describe students who had grown up with digital technologies and have an instinctive understanding of digital technologies [8], and the selected sources of evidence identified this concept as being a barrier to nursing informatics education in undergraduate nursing programs [66,82,107,122]. Lam et al. [122] (p. 306) noted the assumption that healthcare students “may not require explicit, formal education in ICT, entering university with the knowledge and skills to successfully integrate ICT into the healthcare contexts”, with Fosters and Sethares [107] cautioning that whilst undergraduate nursing students may be skilled in the use of technology, this does not take into account the need for information literacy and higher level informatics principles. These findings were reflected in the selected sources of evidence [22,72,82,88,89,90,119]. This issue was still evident in the subsequent review [88], with Raghunathan et al. [126] (p. 1) noting that undergraduate nursing students identified confidence in the use of digital technologies, but that “nursing students’ preparedness for digital health was sub-optimal”. It was noted that the terms ‘digital literacy’ and ‘informatics competency’ were used interchangeably in the literature at times. However, as described by Hariyati et al. [129], digital literacy is an overarching level of competency, which nurses must have to use digital technologies, whereas informatics competency is a higher-order, domain-specific application of this knowledge. The digital literacy of faculty was also identified as a significant issue, with a lack of digital competency, resistance to change and technological stress; this is further addressed in Section 3.3.6.

#### 3.3.5. Evolving Nature of Nursing Informatics

The evolving nature of nursing informatics was identified as a barrier to nursing informatics education in undergraduate nursing programs [23,66,78,81,82,92,97,98,101,102], and it was reflected in the emergence of new tools and new informatics principles. The rapid introduction of new healthcare technologies was identified as an obstacle in embedding these technologies into nursing education [23], with faculty not always understanding definitions of these key concepts or how to apply them to the curriculum [97]. This complexity was further highlighted by Chauvette et al. [81] (p. 7), who noted that nursing informatics “is an elusive concept complicated by the evolving complexity of the digital tools that nurses are expected to use in the clinical environment”. The subsequent review noted that rapid changes in digital health technologies continue to create challenges in embedding nursing informatics into undergraduate nursing programs [84,121,126].

#### 3.3.6. Faculty Responses to Nursing Informatics

The selected sources of evidence identified faculty resistance and technological stress as barriers to nursing informatics education in undergraduate nursing programs [66,80,85,97,100,105,106,107,109,110,118]. Changes to traditional modes and methods of teaching, a lack of understanding of digital technologies and informatics [63,66,80,85,97,98,105,107,109,110,118], a lack of acceptance of nursing informatics [100], and a lack of digital competence [30] were identified as contributors to faculty resistance and technological stress. Burke and Ellis [100] (p. 46) noted that the rapid change of technologies required in clinical settings was correlated with technostress, which was defined as “the inability of an individual to adapt to the use of new technology and to cope effectively with technology”, as first described by Brod [130]. The issues of faculty resistance and technological stress were linked with a lack of best practice guidelines for undergraduate nursing informatics education and limited faculty educational opportunities [105,106,107]. However, in the subsequent review, the focus on attitudes to technologies or faculty resistance was not specifically addressed, suggesting an acceptance of the changing nature of healthcare delivery.

#### 3.3.7. Nursing Informatics Competencies

The selected sources of evidence identified a lack of fit-for-purpose nursing informatics competencies as a barrier to nursing informatics education in undergraduate nursing programs. A lack of contemporary informatics competencies, tailored for purpose, was identified as a barrier to learning [71,74,81,118,119], with digital competencies identified as enabling safe, effective and efficient healthcare within the digital healthcare environment [66,80]. This issue was highlighted by Honey et al. [30], who noted that whilst competency standards for nursing informatics have been developed globally, these competencies have not been adequately adopted within undergraduate nursing education. Similarly, a lack of relevant competencies was identified in the subsequent review with Raghunathan et al. [126] (p. 8) highlighting that “the absence of national entry-to-practice informatics competency guidelines complicates efforts in standardisation of curricula to ensure consistent graduate preparation”.

#### 3.3.8. Nursing Informatics Resources

Limited recommendations, guidelines and evidence-based educational strategies for the integration of Informatics content into undergraduate programs were identified in the review [29,65,80,102,105], with Cummings et al. [66] (p. 331), indicating that “standards, guidelines, and codes of conduct regarding access and use of digital technology in healthcare environments have been outpaced”. The subsequent review also noted that a lack of resources to effectively integrate nursing informatics into undergraduate nursing curricula was identified as an ongoing issue [41,98,121,126], with Zhao et al. [78] identifying the need to develop resources to promote digital health competency in nursing education.

### 3.4. Enablers

The selected sources of evidence identified enablers to the effective integration of nursing informatics into undergraduate nursing education and the development of nursing informatics competency. Enablers were defined as those factors that supported this integration and included integration of digital health technologies into the curricula [79,80,95,96,97], with integration of nursing informatics across the undergraduate nursing curriculum [23,67,74,76,87,95,97,101,102,107,112,115,116,117,118] or as standalone assessments or programs [21,41,71,79,80,91,108,109,110,111,113,114,116]. The importance of standardised definitions of technologies [118], faculty engagement [97], and the use of super-users of informatics to support integration [78,81,92,98,110] was highlighted. The need to assess baseline student digital literacy was also evident [74,82,107,111,113], with McGregor et al. [74] (p. 96) stating “Our current students may indeed be hardwired for digital encounters, but they still require quality learning and practice opportunities to ensure that they develop or translate skills and knowledge to effectively practice with emerging technologies in evolving digital workplaces”. Enablers identified in the subsequent review demonstrated the rapidly involving nature of nursing informatics with a focus on Artificial Intelligence (AI) with predictive analytics and natural language processing [41,64,84,98,103], real-world digital health partnerships [64,78,88,103], and increased exposure to emerging technologies [41,83,84].

## 4. Discussion

Computers were first introduced to nursing care in the 1950s [1,14,15] with Hovenga [34] (n.p.) explaining that nursing informatics in Australia first began around 1984 and has a “torturous history”. This history is evident in contemporary literature, more than 40 years later, with nurses still not being adequately prepared to use digital health technologies [131,132,133,134], due to a lack of consensus on informatics terminologies, including standardised terminologies reflecting nursing practice [135,136,137], a lack of consensus on nursing informatics competencies evidenced by the wide range of current competency standards [138,139,140], and limited undergraduate nursing education regarding nursing informatics [40,141,142]. Concerns regarding the workplace readiness of Registered Nurses continue to be evident, with Kleib et al. [132] (p. 98) stating that new graduates are “exiting undergraduate programs with deficient knowledge in core informatics competencies needed in the workplace and have limited confidence in using DH (Digital Health) tools such as electronic health records” and Kavanagh and Sharpnack [143] (p. 3) indicating that “As educators, we must address the brutal facts of failing to prepare graduates as residency-ready and confront the issue that the academic, or preparation-to practice gap, is increasing despite current efforts”.

The benefits of nursing informatics identified in this scoping review are consistent with global contemporary research [33,77,144]. These benefits include improved patient care outcomes through rapid and systematic patient assessment, reduction in clinical errors, evidence-based practice, and decision-support, clinical reminders, and improved data management. Further benefits include improved time management, cost-effectiveness and improved resource management and improved interdisciplinary communication with the ability for patients/consumers to self-monitor and increasing healthcare accessibility. These outlined benefits demonstrate the growing need for nurses to engage with digital technologies and develop digital proficiency and competency, thereby having the capacity to meet contemporary healthcare challenges [52,132,145].

The benefits of nursing informatics in undergraduate nursing education include recognition of the importance of nursing informatics, development of digital literacy, understanding the importance of data, and access to evidence-based materials with the development of critical thinking and clinical reasoning. The integration of theory and practice, through the use of digital health technologies in academic and clinical settings, is linked with the development of workplace readiness for graduates [84]. Underpinned by the need for digitally capable registered nurses must be the understanding that the development of nursing informatics competency in undergraduate nursing education, not only prepares nursing students for the use of digital health technologies in the clinical setting, but supports digital literacy, clinical reasoning, and evidence-based practice [67].

Contemporary literature has identified a lack of nursing informatics education in undergraduate nursing programs [146,147,148], with Peltonen et al. [149], in The current state of Nursing Informatics—An international cross-sectional survey, explaining that nursing informatics education is failing to meet clinical practice demands and that international guidelines on nursing informatics education are needed to develop nursing informatics competency. Barriers to the integration of nursing informatics into undergraduate nursing education were highlighted in this scoping review. These barriers are (1) a lack of understanding of nursing informatics, (2) the digital literacy of students and faculty, (3) infrastructure and resources, (4) student access to digital technologies, (5) the evolving nature of nursing informatics, (6) faculty responses to nursing informatics, and (7) nursing informatics competencies and resources, and are detailed below.

The selected sources of evidence identified a lack of understanding of nursing and health informatics, limited understanding of nursing informatics applications, and a lack of consistent taxonomy and language related to nursing informatics as barriers to the effective integration of nursing informatics into undergraduate nursing programs. This is evident in contemporary literature with issues surrounding a lack of consistent taxonomy [10,25], resulting in a lack of consensus for practice, education, and research strategies. Similarly, despite evidence of the benefits for patient outcomes [25,140,141], previous studies have identified concerns that nursing informatics may intrude on the traditional role of nursing [150,151] and limit clinical reasoning skills [152,153], thereby detracting from therapeutic communication with patients and their families [154]. This demonstrates the challenges in the current understanding of nursing informatics and “If the purpose of nursing informatics is to improve the safety and quality of patient care, then as a profession, nurses need to be provided with a clearer understanding of nursing informatics” [10] (p. 111). To effectively address the issue of inconsistent informatics’ taxonomy and language, it is recommended for global definitions of nursing informatics and associated fields to be established via means of a global resource. This would provide educators and nurses with a consensus of definitions, which would inform curriculum development and support clinical knowledge.

Digital literacy variability and the belief in the digital native were also identified as a pervasive issue. As addressed by Reid et al. [8] (p. 584), in Challenging the Myth of the Digital Native: A Narrative Review, “the myth of the Digital Native presents a challenge to educators and curricula alike, as exposure to digital technologies does not necessarily equate with digital literacy”. Harerimana et al. [67] (p. 535) also indicated that “Nursing students are assumed to be technologically savvy” but noted that this did not reflect the reality identified in contemporary nursing research. Stunden et al. [77] (p. 10) observed that “Australian nursing students lack the required digital literacy and ICT skills to cope with the everchanging innovative trends in technology” and recommended the development and integration of a national digital literacy framework. To effectively use digital health technologies and associated data, baseline digital literacy is a fundamental requirement. The digital literacy of students and faculty must be evaluated with appropriate remediation provided as needed and supported by the development and integration of a national digital literacy framework into undergraduate nursing curriculum.

Barriers associated with infrastructure included computer crashes, power outages, hardware and software not working, a lack of technical support and poor infrastructure, poor internet connectivity and speed, and the development, cost and maintenance of hardware and software. These technological barriers are particular evident in Australia’s rural and remote regions with gaps in digital connectivity and infrastructure resulting in a digital divide [155] and with “access to reliable, high-quality digital infrastructure across Australia’s diverse geography (remaining) one of the most persistent challenges to achieving digital inclusion” [156]. The Australian Government is committed to the delivery digital services for all people by 2030 [157], with the Tertiary Education Quality and Safety Agency (TEQSA), under the *Higher Education Standards Framework (Threshold Standards) 2021* (Cth) requiring that facilities and infrastructure are “fit for their educational and research purposes” with “secure access to digital information and communication services…continuously available for all users” [158]. Whilst laudable, this commitment is not reflected in access to the necessary digital health technologies for undergraduate nursing education in Australia. Therefore, it is recommended that this deficit be addressed by TEQSA in promoting the digital health competency of undergraduate nursing students.

Limited availability of devices, software and associated resources were further complicated by university and clinical setting policies regarding student use of technologies. Concerns regarding the legal and ethical implications of undergraduate nursing students accessing patient information further impacted integration of nursing informatics into undergraduate nursing education with Grosman-Rimon and Wegier [159] (p. 5) cautioning that “to maximize the potential for health technology benefits, awareness of safety risks and ethical concerns should be increased, and the use of appropriate strategies and measures should be considered.” Therefore, appropriate strategies to address ethical and legal practices must be embedded in the curriculum, including the linking of the bioethical principles with safeguarding of patient data and the professional legal obligations of nurses as addressed in legislation and nursing codes and standards.

The evolving and rapid development of new healthcare technologies was identified as a barrier, due to faculty not always understanding definitions of these key concepts or how to apply them to the curriculum and this was reflected in reports of faculty resistance and technological stress. These issues were linked with a lack of best practice guidelines for undergraduate nursing informatics education, in particular a lack of fit-for-purpose nursing informatics competencies, recommendations, guidelines, and evidence-based educational strategies. To address the lack of resources and best practice guidelines, it is recommended that a national open access repository for nursing informatics education be developed to support the integration of nursing informatics into undergraduate nursing education and support the professional development of university faculty.

As previously stated, Raghunathan et al. [126] (p. 8) noted that “the absence of national entry-to-practice informatics competency guidelines complicates efforts in standardisation of curricula to ensure consistent”. More broadly, approaches to competency standards, education requirements and education vary globally, and there is currently no systematic approach for the implementation of nursing informatics education, leading to potential threats to safe nursing practice [140]. The push to improve nursing informatics skills started appearing in 1995 with the publication of the American Nurses Association’s Standards of Practice for Nursing Informatics [160]. Since this time, initiatives for nursing informatics frameworks have included Technology Informatics Guiding Education Reform Initiative (TIGER) competencies, the American Nurses Association (ANA) competencies, the Quality and Safety Education for Nurses (QSEN) competencies and the Canadian Association of Nursing Schools (CASN) competencies. Despite the development of competency standards for nursing informatics, these competencies have not been adequately adopted and taught within undergraduate nursing education [30]. In addition, a lack of university faculty digital health competency is aligned with resistance to change and technological stress; therefore, digital health competency standards for educators must be developed and embedded as a requirement for employment. In response to these deficits, national digital health competency standards must be developed to inform university faculty competency and support the integration of nursing informatics into undergraduate nursing education. The development and implementation of these competency standards will provide a means by which undergraduate nursing informatics education can be more appropriately evaluated.

Finally, the enablers for the integration of nursing informatics into undergraduate nursing education included the use of digital health technologies in academic and clinical placement settings with exposure to emerging technologies and the development of digital literacy. The integration of nursing informatics content, included throughout the curriculum, across several assessments or as a standalone topic or assessment were identified as key strategies. Curriculum integrated content was viewed as building on existing knowledge but requiring significant faculty engagement and individual assessments highlighted as reducing resource usage but limiting ongoing development of informatics competency. In 2015, Cummings et al. [29] (p. 332) recommended the horizontal and vertical integration of nursing informatics throughout the undergraduate nursing degree, emphasising the need to “normalise NI [nursing informatics] as integral to core nursing activities”. Ten years later, recognition of the context specific applications of nursing informatics, that exist across a range of clinical settings, requires a nuanced approach, which can “more effectively bridge the gap between educational preparation and practice requirements in nursing informatics [144] (p. 2). Contemporary approaches include design-based learning with authentic learning experiences [144], integration of global nursing informatics frameworks to support curriculum development [161,162], and a national curriculum for nursing informatics [163], with Kleib et al. [84] (p. 10) recommending a focus on baseline nursing informatics competency, which can respond to the “dynamic nature of the healthcare system”. Contemporary literature provides a range of enablers for the integration of nursing informatics into undergraduate nursing curriculum [162,164], including through online education [165], simulation [166,167], academic versions of digital health technologies [168], Design-Based Learning (DBL) approaches [144], use of emerging technologies [169], multidisciplinary design [170], and international collaboration [171]. It is self-evident that students must have access to a range of digital health technologies to develop competency and this must not be the sole responsibility of clinical placements; therefore, it is recommended that relationships be established between vendors, healthcare facilities and universities to provide fit-for-purpose digital health technologies to develop student competency with a range of digital health technologies.

### Limitations

The potential limitations of this study included the exclusion of sources of information outside of the time parameters of 2015–2025, those sources not published or translated into English, and sources from countries, which have different regulatory approaches, standards for education and registration, processes, and procedures for the registration of nurses to Australia. These exclusion criteria were used to provide contemporary findings relevant to the Australian healthcare context. However, it is acknowledged that seminal sources of information may have been omitted due this methodological decision. In addition, the desktop review used Scopus only. This was in response to the searches for the initial scoping review, where Scopus provided the highest research results with duplicates of these results evident in the other databases. The grey literature was not searched for the desktop review; this was in response to the searches for the initial scoping review, where the grey literature searches resulted in only 13 sources of information and provided negligible insights. It is acknowledged that limiting the desktop review to Scopus and not including a grey literature search may have resulted in seminal sources being omitted.

## 5. Recommendations

To address the effective integration of nursing informatics into undergraduate nursing education a series of strategies are recommended:

- Global definitions of nursing informatics and associated fields must be established; a global resource would provide educators and nurses with invaluable information to inform nursing informatics education and ongoing professional development. Within the Australian context, consensus of definitions should reflect current definitions supported by the Australian Digital Health Agency and the Australasian Institute of Digital Health, the peak bodies responsible for digital health in Australia.
- The development of university faculty digital health competency must be strengthened through the adoption of competency standards for educators, prioritised by the relevant professional nursing bodies, including the Australian Nursing and Midwifery Accreditation Council, the Nursing and Midwifery Board of Australia, the Australian College of Nursing, and the Australian Nursing and Midwifery Federation, and embedded as a requirement for employment.
- Development of a national open access repository for nursing informatics education would be a useful means of limiting siloed information and providing nurse educators with the opportunity to engage in self-directed professional development.
- It is essential that students have access to a range of digital health technologies. Whilst it is noted that the associated costs of these resources can be prohibitive, it is insufficient to solely rely on clinical settings to provide students with access to digital health technologies. Therefore, relationships should be established between key stakeholders, including vendors, healthcare facilities and universities, to provide fit-for-purpose digital health technologies, with a view to the sharing of resources and providing access to students within the safety of a simulation environment.
- The development of undergraduate nursing curricula be explicitly linked with relevant competency standards, against which educators and universities can measure their own competence and delivery of appropriate information. In the Australian context, this requires the Australian Nursing and Midwifery Accreditation Council to develop nursing informatics competency standards to inform the development of undergraduate nursing programs in Australia. In this way, the theoretical underpinnings of nursing informatics education can be more appropriately evaluated.
- Concerns regarding the digital literacy of students and faculty must be addressed to ensure that effective use of digital health technologies and associated data is realised. Therefore, baseline evaluation of digital literacy for students and faculty, with appropriate remediation provided as required, is an essential requirement and should include the embedding of a digital literacy framework.

## 6. Conclusions

This scoping review highlights the ongoing challenges with the integration of nursing informatics into undergraduate nursing curriculum, focusing on the benefits of digital health technologies both in the preparation of the graduate workforce and in the development of digital literacy, clinical reasoning, and evidence-based practice. Underpinning the drive for ongoing reformation of nursing education are barriers and enablers, many of which have remained largely unchanged over the past 10 years. These findings have provided a snapshot of the nursing profession’s progress in adopting nursing informatics into contemporary practice. As identified, nurses, despite being the largest workforce within healthcare, are still not being sufficiently prepared to use nursing informatics. The benefits of nursing informatics to patient care through rapid access to crucial patient data, systematic patient assessment, less clinical errors, evidence-based practice, cost-effectiveness, and improved patient outcomes and safety are also not being effectively realised. In Australia, since 1984, the importance of nursing and the use of technologies has been highlighted. However, more than 40 years later, registered nurses and undergraduate nursing students are still not being adequately prepared for the contemporary workforce. Undergraduate nursing curriculum must prioritise the digital literacy of students, development of nursing informatics competency standards to inform the development of undergraduate nursing programs, and investment in digital health technologies, if nursing is to leverage the benefits of nursing informatics in the coming decade.

## Figures and Tables

**Table 1:** Exclusion criteria.

Exclusion Criteria	Scoping Review Total Articles *n* = 3303	Subsequent Desktop Review Total Articles *n* = 1555	Total Sources Excluded (*n* = 3895)
Duplicate removal	*n* = 833	*n* = 68	*n* = 901
**First Pass—Title and Abstracts screened**			
Non-nursing	*n* = 656	*n* = 369	*n* = 1025
Non-informatics	*n* = 512	*n* = 299	*n* = 811
Non-nursing education	*n* = 569	*n* = 143	*n* = 712
Not undergraduate curriculum	*n* = 0	*n* = 37	*n* = 37
No curricula recommendations	*n* = 184	*n* = 184	*n* = 368
Limited curricula recommendations	*n* = 61	*n* = 61	*n* = 122
Thesis	*n* = 3	*n* = 0	*n* = 3
Testing of technology or instruments	*n* = 115	*n* = 57	*n* = 172
Organisation documents	*n* = 98	*n* = 0	*n* = 98
Editorials/Opinion	*n* = 127	*n* = 6	*n* = 133
Conference panel	*n* = 15	*n* = 1	*n* = 16
Research proposals	*n* = 4	*n* = 0	*n* = 4
Unable to access document	*n* = 11	*n* = 16	*n* = 27
Not within date parameters	*n* = 1	*n* = 0	*n* = 1
**Second Pass—Full text articles screened**			
Non-nursing	*n* = 0	*n* = 8	*n* = 8
Non-informatics	*n* = 3	*n* = 19	*n* = 22
Non-nursing education	*n* = 1	*n* = 62	*n* = 63
Not undergraduate curriculum	*n* = 13	*n* = 74	*n* = 87
No curricula recommendations	*n* = 4	*n* = 0	*n* = 4
Limited curricula recommendations	*n* = 32	*n* = 33	*n* = 65
Testing of technology or instruments	*n* = 0	*n* = 7	*n* = 7
Editorials/Opinion	*n* = 0	*n* = 5	*n* = 5
Conference panel	*n* = 0	*n* = 1	*n* = 1
Research proposals	*n* = 0	*n* = 2	*n* = 2
Not generalisable to Australian healthcare system	*n* = 15	*n* = 69	*n* = 84
Unable to access document	*n* = 0	*n* = 12	*n* = 12
Not available in English	*n* = 0	*n* = 3	*n* = 3
Not within date parameters	*n* = 0	*n* = 1	*n* = 1
Duplicate	*n* = 0	*n* = 2	*n* = 2
**Data extraction**			
Articles for data extraction	*n* = 46	*n* = 16	*n* = 62

## Data Availability

The original contributions presented in this study are included in the article/Supplementary Material. Further inquiries can be directed to the corresponding author.

## Supplementary Materials

The following supporting information can be downloaded at: https://www.mdpi.com/article/10.3390/nursrep16020042/s1, Table S1: PRISMA ScR Checklist; Table S2: Data extraction table.

## Public Involvement Statement

No public involvement in any aspect of this scoping review.

## Guidelines and Standards Statement

This manuscript was developed using the PRISMA-ScR Checklist [48] and the JBI Manual for Evidence Synthesis [38] in adherence with an a priori Scoping Review Protocol [46]. A complete list of reporting guidelines can be accessed via the equator network: https://www.equator-network.org/reporting-guidelines/prisma-scr/ (accessed on 9 November 2025).
