# Supplementary material for: Nursing Informatics and Undergraduate Nursing Curricula: A Scoping Review
Source: Nurs Rep. 2026 Jan 27;16(2):42. doi: 10.3390/nursrep16020042 (PMC12943083; doi:10.3390/nursrep16020042)
Supplement: Supplementary file 1 [file nursrep-16-00042-s001.zip › Table S2.pdf]

Authors<br>Year	Country	Benefits of Nursing Informatics (NI) education	Barriers to NI education	Enablers to NI education	Faculty development
Almond (2025a)<br>*Health Informatics Education Case Study: A Graduate Nurse Perspective*<br>[1]	Australia	Digital health and informatics are integral to healthcare.<br>• Integrating theory and practice<br>• Safe, quality patient care<br>• Workplace readiness of graduates	Despite theoretical knowledge, graduates indicated low confidence in applying digital health concepts in practice.	EMR navigation, clinical decision support (CDS), and telehealth across curriculum.<br>AI including predictive analytics and natural language processing.<br>Simulation scenarios linking to metropolitan and rural healthcare challenges.<br>Workplace readiness modules to prepare students for interdisciplinary collaboration, data security, and adaptability in healthcare settings.<br>Students to engage in real-world informatics problem-solving in partnership with healthcare organisations.<br>Alumni provided with opportunities to update their digital health competencies through ongoing training.	Not described.
Almond (2025b)<br>*Health Informatics Education Case Study: A Nurse Educator Perspective*<br>[2]	Australia	Educator preparedness is essential for successful delivery of digital health and informatics into curricula.<br>• Practice using technologies<br>• Workplace readiness of graduates	Faculty development remains a barrier to effective integration.	Educator preparedness bridges gap between academic training and clinical application, ensuring graduates are equipped for digital healthcare.	Professional Development through workshops, certifications, and hands-on training with digital health tools.<br>Mentorship and Peer Support.<br>Partnerships with healthcare technology firms to ensure curriculum relevance.<br>Institutional investments in simulation facilities, conference funding, and continuous learning incentives.<br>Continuous assessment of faculty training initiatives to align with educational and clinical needs.
Angel et al. (2016)<br>*Integrating Bar-Code Medication Administration Competencies in the Curriculum: Implications for Nursing Education and Interprofessional Collaboration*	United States of America (USA)	Bar-Code Medication Administration (BCMA) education is essential for contemporary nursing practice.<br>• Practice using technologies	Cost of technologies.<br>Computer crashes, power outages, bar codes not readable.<br>Staff training.<br>Over-reliance on BCMA system to identify errors with reduction in critical thinking.	Described program of BCMA use through 3 semesters using simulation.	Not described.

[3]					
Baxter & Andrew (2018)<br>*Successful Integration of an Academic Electronic Health Record into the Curriculum of an Associate Degree Nursing Program*<br>[4]	USA	Students need exposure to electronic documentation and data management for successful employment after graduation.<br>• Authentic, real-world experience<br>• Practice using technologies<br>• Safe, quality patient care	Internet connectivity.<br>Lack of faculty prioritisation of (Electronic Health Record (EHR).<br>Lack of student acceptance of need to learn EHR; many believed they would not use EHR until after graduation.<br>Barriers to EHR use in clinical setting.	Staggered implementation of academic EHR.<br>End of semester surveys.<br>Modification of academic EHR following discussions with vendor.	Faculty super users.
Bonnel et al. (2018)<br>*Teaching with Technologies in Nursing and the Health Professions: Strategies for Engagement, Quality, and Safety*<br>[5]	USA	Teaching and learning about data management are key requirements for safe, quality nursing care.<br>• Data management<br>• Decision support<br>• Development of critical thinking and clinical reasoning<br>• Development of digital literacy<br>• Ethical use of technologies<br>• Interdisciplinary communication<br>• Patient self-monitoring/ care<br>• Practice using technologies<br>• Safe, quality patient care	Variation of EHRs across schools of nursing.<br>Curricular integration.<br>Faculty acceptance/ approval.<br>Evolving nature of EHRs<br>Development, cost and maintenance of academic EHRs.<br>Clinical practice access.<br>Student computer competencies.<br>Access to EHRs.<br>Patient confidentiality and information security.	Faculty engagement.<br>User-friendly EHRs in non-clinical topics.<br>Clinical EHRs on placement and EHRs in lab sessions.<br>Described assignments linked with NI.	Professional development.<br>Faculty super-users.<br>Address assignments to engage students with EHR data, use of technologies in healthcare systems and populations, leadership and policy issues.<br>Introduction to big data and specific national competencies that are addressed in curriculum.
Booth, Sinclair, Brennan et al. (2017)<br>*Developing and implementing a simulated electronic medication administration record for undergraduate nursing education: Using socio technical systems theory to inform practice and curricula*<br>[6]	Canada	Opportunity for faculty and students to address an immediate learning requirement of modern nursing practice.<br>• Authentic, real-world experience<br>• Practice using technologies	Limited development of eMAR into curricula.<br>Potential to generate new types of medication errors, human-technical interface difficulties & redefining of workflow & administration processes.	SMART eMAR developed.<br>Decision support (commonly found in eMAR technology) added to system, including color-coded (i.e. green or red) prompts for correct or incorrect barcode scans.	Training materials for clinical staff.<br>Academic literature.
Booth, Sinclair, Strudwick et al. (2017)<br>*Strategies Through Clinical Simulation*	Canada	BCMA/ eMAR platforms are increasingly underpinning medication administration processes in contemporary nursing practice.<br>• Authentic, real-world experience	Lack of knowledge regarding implementing BCMA and eMAR into curricula.	Real and simulated BCMA/ eMAR.<br>Homegrown or Open Source BCMA/eMAR Platform.	Safe practices seminar.<br>Professional development.

to Support Nursing Students and Their Learning of Barcode Medication Administration (BCMA) and Electronic Medication Administration Record (eMAR) Technologies<br>[7]		• Practice using technologies	BCMA/eMAR can cause new types of medication errors are unlikely within a paper-based administration process.<br>Students confident using technology to support medication administration but fearful of committing a serious error.<br>Prohibitive costs of BCMA/ eMAR.<br>Technical difficulties.<br>Lack of faculty knowledge.<br>Lack of student knowledge of medication rights.	Purposeful and carefully attenuated use to avoid cognitive overload.<br>Embedding clinical reasoning into use of technology.<br>Consider cost and sustainability of the system.	
Bove (2023)<br>*Nursing Faculty Informatics Competencies*<br>[8]	USA	NI is an essential requirement for safe and efficient patient care and faculty with NI competence can help students advance the use of technology and informatics at the bedside.<br>• Data management<br>• Development of digital literacy<br>• Effective time management<br>• Safe, quality patient care	Lack of understanding of what informatics is and how to integrate it into the curriculum.<br>Lack of resources.<br>Limited professional development.<br>Lack of standard criteria when teaching NI competencies, although there is agreement that nursing informatics competencies are an important part of curricula.	Development of basic computer skills.<br>Partnering with faculty with informatic expertise.<br>Training on informatics.<br>Use of analytics.	Use of AACN, NLN and informatics organisations tools.
Brunel & Allert (2025)<br>*Integrating Experiential Informatics and Quality Improvement in Baccalaureate Education*<br>[9]	USA	Current education tends to focus on theory, but students need access to practical experiences in using informatics technologies.<br>• Data management<br>• Development of digital literacy<br>• Ethical use of technologies<br>• Evidence-based practice<br>• Integrating theory and practice<br>• Practice with technologies<br>• Quality assurance activities<br>• Safe, quality patient care	Current education typically focuses on theory, terminology, and an overview of systems, with burden of practical education on clinical partners	Professional practice strategies outlined by the Institute for Healthcare Improvement.<br>Theoretical and practical elements of nursing informatics.<br>Use of clinical practice councils with auditing of nursing charts.<br>Scaffolded modules.	Not described.
Brunner et al. (2018)<br>*An eHealth Capabilities Framework for Graduates and Health Professionals: Mixed-Methods Study*<br>[10]	Australia	Developing a eHealth capable workforce is becoming a key priority with expectation eHealth ready graduates.<br>• Cost-effectiveness<br>• Data management<br>• Ethical use of technologies<br>• Interdisciplinary communication<br>• Patient communication<br>• Rapid health assessment<br>• Workplace readiness of graduates	Not described.	Further research exploring the implications for the existing health care workforce, with focus on identifying the relevance & impact of capability statements on policy and practice, including recruitment, professional development, performance management and systems improvement activities•	Not described.

Burke & Ellis (2016)<br>*Electronic Health Records: Describing Technological Stressors of Nurse Educators*<br>[11]	USA	Graduates should be competent in the use of EHRs with links to quality of patient care, reduced costs and improved communication.<br>• Data management<br>• Decision support<br>• Practice using technologies	Technological stress.<br>Lack of student access to EHR training materials.<br>Need for educators to learn new EHRs<br>Lack of student knowledge of EHRs.<br>Lack of EHR support.<br>Barriers to EHR use in clinical setting.<br>Cost prohibitive commercial EHRs.<br>Different EHR systems makes preparing students difficult.	Students need to be taught use of health information systems and EHRs.<br>Acquisition of EHRs for academic setting.<br>Development of cost-effective access to range of EHRs for academic setting.<br>Use of EHRs in academic setting to reduce costs and time in clinical setting.<br>Assigning of students to one clinical setting for entire semester to increase familiarity with clinical EHR.	Partnerships with clinical facilities to support access to EHRs.<br>Professional development.
Chauvette, Kleib & Paul. (2022)<br>*Developing nursing students' informatics competencies - A Canadian faculty perspective*<br>[12]	Canada	Intentional integration of NI into curricula supports students to develop knowledge, skills, and attitudes necessary for safe nursing practice.<br>• Development of digital literacy<br>• Evidence based educational resources	Learning in a digital-paper environment.<br>Limited and timely access in clinical environment.<br>Students do not have access to digital tools due to the limited number of computers, lack of log-in credentials to access computers and access to Wi-Fi in clinical settings.<br>Some clinical environments continue to ban use of mobile devices which limits experiential learning opportunities.<br>Evolving complexity of NI tools.	Faculty reported using a health science librarian to assist students in developing NI competency.<br>Librarians support students in searching and critically appraising the literature, ensuring that the information is reliable and high quality as well as relevant to nursing practice.	Minimum NI competencies for faculty.
Chauvette, Paul & Kleib (2022)<br>*The impetus of COVID-19 in transforming nursing education through informatics*<br>[13]	Canada	Nursing curricula and teaching strategies need to teach with and about technology to better inform healthcare interventions that improve healthcare outcomes and prepare the nursing workforce.<br>• Development of digital literacy	Integration of digital tools into curricula and teaching.<br>Varying levels of digital literacy in students.	Faculty integrating digital tools into education.<br>Integration of digital tools and informatics to increase informatics capacity amongst nurses.<br>Faculty use of nursing informatics self-assessment tools.<br>Use of EHRs.	Faculty engagement and professional development are critical<br>components for the successful implementation of curricular changes.
Clancy (2015)<br>*The Use of Anatomical Side Markers in General Radiology: A Systematic Review of the Current Literature*<br>[14]	USA	Rapid growth of digital health technologies has created a complex healthcare system and education needs to address the use of patient-care technologies.<br>• Clinical reminders<br>• Cost effectiveness<br>• Data management<br>• Decision support<br>• Ethical use of technologies<br>• Medication management<br>• Patient assessment<br>• Population healthcare	Not described.	Integrated across curriculum and existing courses and programs.<br>Detailed description of BSN Health Informatics (HI).<br>Academic EHRs.<br>Integration of technologies to document patient care.	Not described.

		• Practice using technologies<br>• Systematic patient assessment			
Clever Together. (2015)<br>*A vision for the Changing Faculty Role: Preparing Students for the Technological World of Health Care*<br>[15]	USA	Digital health technologies can enable new patterns of patient care and further inform nursing interventions.<br>• Patient self-monitoring/ care<br>• Safe, quality care<br>• Virtual care	Faculty not experienced with technology.<br>Scant quality educational resources.<br>Nursing education has not kept up with evolving practice requirements, including the focus on innovative technologies.	Instructional designers and informatics specialists to facilitate course design.<br>Learning opportunities to develop technological skills and knowledge.<br>Collaborate with practice partners to increase opportunities for contextual learning by designing clinical encounters using technology and simulation across the continuum of care<br>Learning activities that address shift to public and community health resources.<br>Create clinical experiences for students to assess consumer eHealth literacy and assist patients to translate data for meaningful use.<br>Incorporate health promotion & health maintenance.	Incentive-based programs for faculty.<br>Institutional and financial support for faculty development.<br>Professional development.<br>Identify ways to work efficiently with Information and Communication Technologies (ICT).
Cross & Kendrick (2025)<br>*Artificial intelligence and the meta-paradigm*<br>[16]	USA	Healthcare technology can improve data availability, offload documentation, and improve patient outcomes; therefore, it is important for faculty to be competent in the use of digital health technologies.<br>• Authentic real-world preparation<br>• Data management<br>• Development of digital literacy<br>• Ethical use of technologies<br>• Practice using technologies<br>• Rapid patient assessment<br>• Safe, quality patient care	Lack of consensus on the legal and ethical use of AI, including information ownership.<br>Fear of largely unknown capabilities of AI.	Nurse educators must ensure that students have the professional competencies to deliver nursing care using AI.<br>Focus on art and caring of nursing whilst addressing current AI competencies.<br>Real world preparation.<br>Integration of AI technologies into curricula.<br>Designing of clinical experiences that "recognise" the technology.<br>Maintain clinical partnerships so students are prepared for the clinical workspace.<br>Collaboration with interprofessional academic staff to develop simulations that mimic real world experiences.<br>Discussion focusing on ethical use of AI.<br>Threading of AI throughout curriculum.	Not described.
Cummings et al. (2015)<br>*Teaching Nursing Informatics in Australia, Canada and Denmark*	Canada<br>Australia<br>Denmark	Increased recognition of the importance of NI education - Denmark more developed than Canada & Australia.<br>• Cost effectiveness<br>• Ethical use of technologies<br>• Recognition of importance of NI	Lack of time.<br>Lack of developmental or technical assistance.<br>Lack of faculty knowledge and commitment.	Peer to peer network.<br>Select, access, search and evaluate appropriate databases.	Peer-to-peer network providing mentorship and support.<br>Professional development.

[17]		• Safe, quality patient care	Costs.<br>Lack of training opportunities.<br>Appropriate software.<br>Lack of basic informatics content in curriculum.<br>Limited NI education.<br>Lack of NI competencies in current Australian nursing codes.	Relate information technology, information literacy and evidence- based practice.<br>Define, describe and discuss basics about standardised languages and their impact.<br>Describe transformation of data and information into knowledge management.<br>Introduction to EHR.<br>Handling patient information ethically, data security, social media use and communication•	
Cummings et al. (2017)<br>*Integrating Health Informatics into Australian Higher Education Health Profession Curricula*<br>[18]	Australia	E-health, HI and health should be viewed as synonymous in contemporary healthcare with a growing need to incorporate e-health & HI skills in higher education programs globally.<br>• Data management<br>• Patient communication	Historical ad hoc approach and view of informatics as not part of clinical knowledge.<br>Education sporadic and uncoordinated.<br>Lack of systematic approach to teach, assess, evaluate, or audit HI in professional education.<br>Overloaded curricula.<br>Complex, changing digital environment.<br>Assumptions about digital natives.<br>Barriers using technology on placement.<br>Limited understanding of HI and HI specialisation of HI.<br>Poor understanding of HI amongst many of the health professional education staff•	Mobile computing, networking & digital professionalism training.<br>Classroom and simulation-based activities.<br>Capability to be digitally professional needs to be demonstrated prior to work integrated learning to ensure high quality and safe patient-care.<br>Integrated programs linking HI research and teaching program.<br>HI education and culture across health professional education courses.<br>HI technologies as normal element in education.<br>Discuss legal, political and cultural issues around use of HI.<br>Virtual community of practice using Twitter.	Educators must be prepared to teach students how to become digitally literate and prepare them for their healthcare experiences.
Doerner et al. (2025)<br>*Student growth in informatics competencies after a nursing informatics course*<br>[19]	USA	Healthcare is increasingly more technology-driven, requiring professionals to access, analyse, and implement data-based interventions.<br>• Accessible care<br>• Data management<br>• Decision support<br>• Development of digital literacy<br>• Understanding importance of data	Varying methods of NI education delivery and assessment.<br>Blurring of lines between IT and NI	Use of SANICS (Self-Assessment of Nursing Informatics Competency Scale) to self-evaluate development of NI competency.<br>NI course, introducing students to information management, standardised terminology, electronic health records.<br>Assignments included discussion boards and papers on key informatics concepts, EHR roles, and clinical decision-making tools.<br>Either a standalone course or threaded throughout curriculum.	Not described.

Forman, Armor & Miller (2020)<br>*A Review of Clinical Informatics Competencies in Nursing to Inform Best Practices in Education and Nurse Faculty Development*<br>[20]	USA	Present and future nurses must be able to use informatics and technology to facilitate critical decision-making for optimal patient outcomes.<br>• Data management<br>• Decision support<br>• Rapid health assessment<br>• Safe, quality patient care<br>• Workplace readiness of graduates	No consensus on how competency education is best implemented.<br>Lack of effective educational strategies.<br>Faculty not appropriately engaged with technology.<br>Ongoing lack of integration of Clinical Informatics (CI) education in curricula.	Need for continued research to provide direction about the expected CI competency of graduate nurses and training faculty need to facilitate student learning.<br>Resources available through professional organisations, including HIMSS and NLN.	Not described.
Forman, Flores & Miller (2020)<br>*An Integrative Literature Review of the Use of Electronic Health Records for Clinical Nursing Education*<br>[21]	USA	EHR training can provide a comprehensive understanding of the value technology brings to a patient's quality of care.<br>• Data management<br>• Development of digital literacy<br>• Practice using technologies<br>• Rapid health assessment<br>• Recognition of importance of NI	Cost of EHR.<br>Cost of support personnel, technology upgrades and faculty time.<br>Faculty stress.<br>Lack of technology support.<br>Lack of faculty experience with EHR.<br>Lack of opportunity for EHR training for students and staff.<br>Lack of time.	Practice with EHR and Academic EHR.<br>Use of tools to assess NI competency.<br>Further research into NI integration into curriculum.	Cultivating a positive attitude among faculty needed to improve perception of EHRs in education.<br>TIGER training.
Foster & Sethares (2017)<br>*Current Strategies to Implement Informatics into the Nursing Curriculum: An Integrative Review*<br>[22]	USA	Nurses who are experienced with using technology and databases can retrieve information to make sound decisions based on current research rather than opinion.<br>• Data management<br>• Ethical use of technologies<br>• Evidence-based practice<br>• Safe, quality patient care	Lack of faculty competence.<br>Lack of faculty awareness of NI curricular guidelines.<br>Lack of consensus on integration of NI into curriculum.<br>Inconsistent infusion of NI knowledge and skills into education.<br>Belief in Digital Native.	Baseline informatics competencies by computer- generated, computer-graded assessment tools; using results to prioritise what informatics strategies should be included in curriculum.<br>Four major content areas - professional responsibility, care delivery, community nursing and care of populations and leadership/ management faculty.<br>Case scenarios that increase in difficulty over the course of student's education.	Support from outside personnel and peers.<br>Professional development.<br>Inventory of informatics resources and tools.
Gambo et al. (2017)<br>*Can Mobile Technology Enhance Learning and Change Educational Practice?*<br>[23]	USA	Integration of high-fidelity simulation into nursing curricula gives students the opportunity to practice newly learned skills and reinforces prior knowledge in a safe patient care learning environment.<br>• Data management<br>• Evidence based practice<br>• Safe, quality patient care	Lack of technology support.<br>Internet speed.<br>Cost of software & hardware.<br>Availability of devices.<br>Second career learners.<br>Generational resistance.<br>Changing role of educator.<br>Infection control.<br>Patient privacy.	Real life simulation scenarios.<br>QR code on "patient" wristband with health information.<br>Integration of simulation into all stages.<br>Presimulation preparation.<br>Debriefing.	Baseline assessment of educator technology proficiency.<br>Professional development.,

Hamilton et al. (2021)<br>*The Integration of Telehealth in Nursing Education: A New Frontier*<br>[24]	USA	Use of simulation is one of the most effective methods to enhance telehealth integration in curricula.<br>• Accessible care<br>• Cost effectiveness<br>• Development of critical thinking and clinical reasoning<br>• Patient communication<br>• Practice using technologies<br>• Rapid health assessment<br>• Safe, quality patient care<br>• Workplace readiness of graduates	Lack of funding.<br>Lack of knowledge among faculty.<br>Lack of laboratory support staff.<br>Lack of laboratory space.<br>Lack of time to develop simulated telehealth case studies.<br>Lack of time for hands-on experience with technology.<br>Older technology.<br>Lack of funding to maintain the equipment, hire additional staff and provide training for faculty<br>Lack of telehealth clinical sites.	Telehealth program using equipment with simulated telehealth experience.	Not described.
Harerimana et al. (2022)<br>*Nursing informatics in undergraduate nursing education in Australia before COVID-19: A scoping review*<br>[25]	Australia	Increasing demands for universities to produce digitally competent graduates who can use ICT to deliver quality healthcare.<br>• Decision support<br>• Development of critical thinking and clinical reasoning<br>• Development of digital literacy<br>• Ethical use of technologies<br>• Evidence based practice<br>• Resource management<br>• Safe, quality patient care	Lack of NI guidelines.<br>Non-adherence to standards and criteria for teaching NI.<br>Ambiguity of NI terminology.<br>Clinical simulations - lack of time, technical and academic support, equipment and access to dedicated simulation environment.<br>Lack of guidelines/ frameworks to develop NI and guide integration of technology in curriculum.<br>Students' poor ICT literacy.<br>Limited access to ICT tools and applications.<br>Limited exposure to NI due to lack of interaction with NI in clinical placement, caused by ethical issues to access patient data.	Training packages to improve digital literacy and competencies to teach NI to students.<br>Baseline perspective of how NI was embedded & integrated into nursing education in Australia before COVID-19.<br>NI in undergraduate nursing education recommended by the ANMAC but differs across institutions.<br>Mode of delivery of instructions included online, virtual and blended learning.<br>Technology-supported teaching strategies essential.	Multidisciplinary collaboration & partnership with academic learning support facilitators, unit coordinators, team coordinators and librarians.<br>Presence of specialists in NI and experts in online course development helped faculty develop course content.
Hay et al. (2017)<br>*"iM Ready to Learn": Undergraduate Nursing Students Knowledge, Preferences, and Practice of Mobile Technology and Social Media*<br>[26]	Australia	Nursing academia should teach appropriate use of mobile technology and social media to positively affect use of such technologies in the future nursing workforce.<br>• Evidence based practice	Costs of devices.<br>Lack of wireless access.<br>Infection control.<br>Adherence to hospital guidelines.<br>Challenge for undergraduate nursing course designers is to build on findings to use social media and mobile technology in nursing research and education.	Twitter hashtag for networking, earning and content consolidation.<br>On-campus conferences relating to mobile technology and social media use in nursing.	Committee formed to assist both staff and students to support greater integration of digital technologies within curriculum.

Hern et al. (2015)<br>*Facilitating adoption of informatics and meaningful use of electronic health records with nursing faculty*<br>[27]	USA	Pedagogical generation gap between faculty and students must be addressed to ensure a job-ready workforce.<br>• Data management<br>• Ethical use of technologies<br>• Interdisciplinary communication<br>• Practice using technologies<br>• Safe, quality patient care<br>• Workplace readiness of graduates	Restricted use of EHRs in clinical practicums.<br>Older faculty and clinical nurses tend to be slower to adopt technology.	Organisational responsiveness.<br>Faculty incentives.<br>Expert consultants.<br>State-of-art campus labs.	Pilot with seminars and national consultants' presentations to faculty regarding their knowledge, skills and attitudes about informatics and use of EHRs.<br>Participant faculty members received an iPad as incentive to participate and kept the iPad while employed in the school.
Honey et al. (2021)<br>*Identifying How to Support Nurse Educators Nationally to Teach Nursing Informatics*<br>[28]	New Zealand (NZ)	Preparing students for practice with NI competency is challenging for many nurse educators.	Cost, policy, training, staffing & support.<br>Constantly evolving nature of ICT.<br>Lack of access to devices for students.	Motivated staff engaged with this aspect of nursing education.<br>A health system that supported informatics growth of nursing students.<br>An organisation that dedicated time and resources to NI.<br>National resources.	Professional development for nurse lecturers.<br>Nationally available resources would be helpful.
Hovenga & Grain (2016)<br>*Learning, Training and Teaching of Health Informatics and its Evidence for Informaticians and Clinical Practice*<br>[29]	Australia	There is a need for a framework to inform curriculum and workforce capacity building.<br>• Data management<br>• Ethical use of technologies<br>• Effective time management<br>• Safe, quality patient care	Gulf between ICT research, teaching staff, health research and health professional education.<br>Health workforce generally appears to have limited understanding of HI discipline.<br>Many stakeholders unable to differentiate between ICT skills and HI skills and knowledge.	Frameworks could be used to develop inventory of job roles to assist HI educators with identification of knowledge, professional, technical and behavioural competency needs along with required experience and qualification levels.	Not described.
Kleib et al. (2025)<br>*Education About Digital Health and Artificial Intelligence and Learning Needs: Perspectives of Undergraduate Nursing Students*<br>[30]	Canada	Next generation of nurses must be prepared for digital revolution.<br>• Authentic, real-world experience<br>• Development of digital literacy<br>• Ethical use of technologies<br>• Integrating theory and practice<br>• Practice using technologies<br>• Understanding importance of data	Limited and mostly didactic theoretical content.<br>Limited hands-on experiences with technology.<br>Limited educator capabilities.<br>Negative responses from nurses in clinical settings.	Student self-directed learning and finding opportunities to work with technologies in clinical setting.<br>Clinical learning experiences.<br>Support of nurses in clinical setting.<br>Need for systematic education.<br>Curricula integration of digital health.<br>Increased exposure to clinical applications - existing and emerging.<br>Focus on safe, ethical practice.	Not described.
Kleib et al. (2024)<br>*Digital Health Education and Training for Undergraduate and Graduate Nursing*	Canada	Technology will continue to play a pivotal role in modern-day health care; therefore, it is important that education keep abreast of the rapidly evolving technological revolution.<br>• Accessible healthcare<br>• Development of digital literacy	Significant gaps and limitations in the scope of digital health education at the undergraduate and graduate levels.<br>Lack of a consistent taxonomy.<br>Lack of direction regarding the integration of AI in nursing education.	Enhancing the digital health education should be a policy priority.<br>Foundational knowledge, including core concepts, related to digital healthcare.<br>Augmenting learning with new technologies.	Not described.

Students: Scoping Review<br>[31]		• Ethical use of technologies<br>• Practice using technologies<br>• Safe, quality patient care	Lack of foundational knowledge on digital health in curricula.	Digital technologies integrated into teaching to increase digital competency.<br>Digital health education should be embedded throughout all levels of education.<br>AI competencies.<br>Clinical preceptors.<br>Guidelines for health informatics.<br>EHR simulations.<br>Telehealth educational strategies.	
Kleib & Olson (2015)<br>*Evaluation of an informatics educational intervention to enhance informatics competence among baccalaureate nursing students*<br>[32]	Canada	Quality and safety of patient care are key drivers to improving NI competence in students.<br>• Data management<br>• Decision support<br>• Safe, quality patient care	Student's competing commitments.<br>Lack of interest about NI and perception that NI is not relevant to nursing practice.<br>Limited access to technology applications.<br>Lack of faculty knowledge and skills.<br>Use of conventional approaches.<br>Faculty members equated being involved in distance learning, online learning and web-based instruction with being prepared in informatics.<br>Limited integration of handheld devices.<br>Informatics education in curriculum insufficient for clinical practice.	Development and pilot test of informatics educational intervention.<br>Content - basic HI and NI principles and some tools available through HI applications.<br>Evaluation of efficacy of intervention in increasing knowledge, confidence and attitude outcomes towards the EHR.	Not described.
Lam et al. (2016)<br>*Preparedness for eHealth: Health Sciences Students' Knowledge, Skills, and Confidence.*<br>[33]	Australia	eHealth is linked with efficient and effective care delivery and scaffolded learning is required to support students' competency attainment.<br>Development of digital literacy	Limited understanding of eHealth as it relates to professional practice.<br>Assumption of student eHealth readiness due to being "digital natives".<br>Limited exposure to eHealth within professional practice.<br>Difficulty transferring existing ICT skills to higher education learning context.<br>Females less confident than male counterparts.	Online questionnaire student use of ICT.<br>Faculty-wide initiative to increase access to eHealth with electives including interviews with health professionals.<br>Focus on how and why technology is used for health, including practical role-play sessions to support students' eHealth confidence and skill development.	Faculty wide initiative to increase eHealth experiences.
Lokmic-Tomkins et al. (2023)<br>*Integrating interprofessional electronic medical record teaching in preregistration healthcare degrees: A case study*	Australia	The rapid integration of digital health technology (DHT) into interdisciplinary healthcare amplifies the need for nursing educators to focus on health informatics capabilities.<br>• Data management<br>• Ethical use of technologies<br>• Interdisciplinary communication<br>• Practice using technologies	Lack of educator competency.<br>Financial constraints.<br>Curriculum overload.<br>Constantly evolving technology	A scaffolded pre-registration health informatics curriculum.<br>Interprofessional collaboration with authentic clinical placements that focus on digital health technologies.<br>Adaptive curricula that reflects new and emerging digital health technologies.	Stakeholder support - including educators, clinical placement providers, and health informatics officers.

[34]		• Safe, quality patient care		Implementation strategies for health informatics curricula must align with real-world digital environments, considering students' digital literacy, information skills, and data analytics.<br>Starting with widely used DHTs like electronic medical records and telehealth, the curriculum should evolve to include innovations such as robotics, artificial intelligence, and machine learning.<br>Authentic assessments and realistic case studies are essential to develop critical thinking skills.	
Luo & Kalman (2018)<br>*A technology training protocol for meeting QSEN goals: Focusing on meaningful learning*<br>[35]	USA	Nurses' technological knowledge of skills in and attitudes toward new technologies in the health care setting are critical to improving healthcare outcomes.<br>• Data management<br>• Safe, quality patient care	Nurses who did not grow up in computer age may not have sufficient computer knowledge.<br>End users of technology unable to see "big picture" of how technologies help in collecting, recording, protecting, storing, utilising, analysing and reporting information and data related to patient safety and improved outcomes.<br>Integration of technologies hampered by nurses' workarounds.<br>Real-workflow culture does not fit design purposes of EHRs.<br>One size fits all training fails to consider prior learning.	Procedure to connect students' prior knowledge with use of new software.	Not described.
Mather & Cummings (2015)<br>*Empowering learners: Using a triad model to promote eHealth literacy and transform learning at point of care*<br>[36]	Australia	Future-proofing health of patients by improving eHealth literacy in situ is an innovation that can no longer be ignored.<br>• Ethical use of technologies<br>• Patient self-monitoring/ care	Few nursing courses overtly describe HI competency level expected by graduates.<br>Lack of clear strategies for integrating competencies into curricula.<br>Lack of investment in tools representative of real-world settings.<br>Students lack competencies for finding and evaluating health information.<br>Students have difficulty discriminating between primary and secondary sources and credible sites for health information.	Use of a triad model using a case scenario - nurse/ patient/ health information.	Need for understanding about eHealth literacy concept.<br>Nursing supervisors must be aware of digital reading habits.
Mather & Cummings (2016)<br>*Issues for Deployment of Mobile Learning by*	Australia	Proficiency in using digital technologies must requires scaffolding through the curricula.<br>• Ethical use of technologies	Resistance by non-technology users.<br>Inappropriate use of technology.<br>Reduced eye contact and barrier with patient interaction.	Embedding use of health technologies and informatics into curricula.<br>Leadership and organisational level support.	Nurse supervisors need to support legitimate use of mobile devices in clinical settings.

Nurses in Australian Healthcare Setting<br>[37]		• Evidence based educational resources<br>• Evidence based practice<br>• Safe, quality patient care	Negative response of other nurses and patients - looks unprofessional.<br>Technical issues - battery life, screen size, availability of charging ports, speed of internet or resources not regularly updated.<br>Risks to patient privacy.<br>Potential theft of device.<br>University and organisational policy not to use mobile devices during placement•		
Mather, Cummings & Nichols (2016)<br>*Social Media Training for Professional Identity Development in Undergraduate Nurses*<br>[38]	Australia	Connect students with peers, colleagues, experts and organisations to assist them to keep up to date with important professional changes and provides opportunities for employment and professional connections.<br>• Evidence based practice<br>• Interdisciplinary communication	Gap in curriculum to ensure appropriate guidance and support in the use of newer platforms.<br>Lack of scaffolded learning about professionalism.<br>Lack of consistency in curriculum topics is confusing for educators, clinicians and students.	Design to enhance professionalism and professional identity online.<br>Management of professional identity.<br>Development of mechanisms to support students with digital platform usage.	Need to integrate and model appropriate social media use in the classroom and during placement.
McGregor et al. (2017)<br>*Preparing E-Health Ready Graduates: A Qualitative Focus Group Study*<br>[39]	Australia	There is a demand for universities to provide eHealth ready graduates who are ready for the workforce.<br>• Data management<br>• Ethical use of technologies<br>• Evidence based practice<br>• Patient self- monitoring/ care<br>• Safe, quality patientcare	Limited understanding of core competencies for eHealth.<br>Need to move focus beyond technical skills and to broader professional competencies.	Focus on technical skills required to practice within digital contexts should be expanded.<br>Reinforce existing competencies.<br>Acknowledge and adapt individual's existing competencies to make them transferable to eHealth contexts.<br>Introduce new learning and provide opportunities for interactions with e-health within education and practice encounters.	Not described.
Mollart et al. (2025)<br>*Student confidence and knowledge with electronic medical records through on-ward simulation: An evaluation study*<br>[40]	Australia	Registered nurses require digital health literacy to use patient electronic medical records with simulation-based training enhancing technical and cognitive skills in students.<br>• Data management<br>• Development of digital literacy<br>• Development of critical thinking and clinical reasoning<br>• Practice using technologies<br>• Safe, quality patient care	Limited utilisation of academic EMR in pre-registration nursing education in the Australian context.<br>Gaps in nursing students' informatics competencies for digital health practice.<br>Limited university clinical workflow knowledge.<br>Lack of EMR licenses.	Use of simulation.<br>Simulation sessions using EMR on clinical placement over 10-week placement.<br>Sessions designed by clinicians.<br>Use of simulated patients.<br>Conducted on ward during shift changeover in afternoon.<br>Debriefs with clinicians.<br>Guided reflection.	No described.

O'Connor & Andrews (2015)<br>*Mobile Technology and Its Use in Clinical Nursing Education: A Literature Review*<br>[41]	United Kingdom (UK)<br>Ireland	Mobile technologies can augment nursing education and provide timely and evidence-based information to support clinical decision-making.<br>• Evidence based practice<br>• Interdisciplinary communication<br>• Safe, quality patient care	Lack of definition and clarity on mobile technology, range of devices and rationales for selection.<br>Technical issues - freezing, crashing etc., lack of Wi-Fi connection, difficulties with small screen.<br>Cost of equipment.<br>Poor computer literacy.<br>Lack of technical support.<br>Lack of tailored resources.<br>Negative attitudes of nursing students and staff.	Drug reference guide the most used software program.<br>Improved students' pharmacological knowledge and patient education.<br>Laboratory and diagnostic manuals to check physiological indicators of disease.<br>Clinical portfolio software.<br>CDS tools.<br>Enhanced clinical knowledge and knowledge retention.<br>Flexible form of education that matches individual's needs.	Nurse educators should consider adopting handheld devices to augment nursing education and practice.
O'Connor et al. (2017)<br>*Time for TIGER to ROAR! Technology Informatics Guiding Education Reform*<br>[42]	UK<br>USA<br>Germany	Technology underpins all three legs of the proverbial stool i.e. education, research and practice, it is critical that nurses are adequately trained in informatics.<br>• Decision support<br>• Evidence based practice<br>• Interdisciplinary communication<br>• Safe, quality patient care	Clinical staff resistant due to limited technical knowledge and capability.<br>Poor digital literacy.<br>Lack of training in clinical setting.<br>Some educators and researchers have been slower to develop technological knowledge and abilities, which means they cannot make the best use of electronic tools and applications in their respective roles.	Sharing of ideas & solutions.<br>TIGER (Technology Informatics Guiding Education Reform) Initiative to guide creation of solutions to suit the needs of local health workforce and population of people they care for.	Not described.
O'Connor & LaRue (2021)<br>*Integrating informatics into undergraduate nursing education: A case study using a spiral learning approach*<br>[43]	UK	Educators must invest in NI education to develop workplace ready graduates.<br>• Data management<br>• Development of digital literacy<br>• Ethical use of technologies<br>• Patient self-monitoring/ care<br>• Safe, quality patient care	Lack of faculty expertise on HI.<br>Lack of consensus on which HI concepts should be taught.<br>Rapidly evolving technology.	Individual learning units corresponding to the six competency domains and learning descriptors are being designed by faculty and integrated into Bachelor of Nursing program.	Professional development.
Pobocik (2015)<br>*Using an Educational Electronic Documentation System to Help Nursing Students Accurately Identify Patient Data*<br>[44]	USA	Integration of Education Electronic Documentation System (EEDS) into curriculum can help students accurately identify patient data and develop critical thinking skills.<br>• Data management<br>• Decision support<br>• Development of critical thinking and clinical reasoning<br>• Evidence based practice<br>• Interdisciplinary communication<br>• Practice using technologies	In some clinical settings students cannot use the EHR.<br>Nurses in practice have negative attitudes about using an Electronic Documentation System (EDS).<br>Poor documentation potentially negatively affects patient care, professional accountability and organisational risk/ nurses who have poor computer skills may inaccurately report patient data.	Development and integration of educational EHR.	Not described.

		• Safe, quality patient care<br>• Understanding importance of data<br>• Workplace readiness of graduates			
Raghunathan et al. (2021)<br>*Use of academic electronic medical records in nurse education: A scoping review*<br>[45]	Australia	Because nurses are at the frontline of care, with key roles in collecting, recording & managing health data, proficiency with ICT and informatics skills is essential.<br>• Data management<br>• Development of digital literacy<br>• Practice using technologies<br>• Safe, quality patient care	Nurse education has delegated proficiency with digital records to clinical settings with students exposed to EMRs during clinical placements.<br>Nursing graduates are inadequately prepared to use digital systems in practice.<br>Lack of curriculum interventions to address health technology capabilities poses risks as inadequate knowledge to operate EMR systems in the healthcare environment can compromise clinical decision-making, patient safety and quality of healthcare outcomes.<br>Individual challenges including issues with product features, software functionality and lack of faculty expertise.	Assignments and learning activities developed to mimic clinical systems.<br>Academic Electronic Medical Records (AEMRs) in skills & simulation laboratories, or classrooms or other settings.<br>Introduction to AEMRs within programs varied and AEMRs were usually embedded within initial fundamentals courses.<br>Essential foundational nursing skills were also embedded into AEMR activities.<br>Range of activities and resources to engage learners with AEMRs.<br>Integrating case scenarios with AEMRs was highlighted as an effective way to assist students to develop skills to confidently use clinical EMRs.	Not described.
Raghunathan et al. (2022)<br>*Utilisation of academic electronic medical records in pre-registration nurse education: A descriptive study*<br>[46]	Australia	Incorporating AEMRs offers practical and meaningful learning experiences and improves education experience and safety of care.<br>• Authentic, real-world experience<br>• Cost effectiveness<br>• Data management<br>• Decision support<br>• Practice using technologies<br>• Rapid assessment	Healthcare is rapidly digitising but application of AEHRs in nurse education not extensive across Australia and New Zealand.<br>Gaps identified in academic-healthcare partnerships and sharing of resources.<br>Cost, lack of funds, lack of technology support and inadequate faculty knowledge.<br>Faculty lack of NI and technology knowledge.<br>Lack of time to develop and teach AEMR content within an expanding nursing curriculum.<br>Lack of data about student preparedness for digital health practice.	AEMRs incorporated into curricula for documentation, health assessment & care planning, nursing notes and reading medical orders, medication charting and interprofessional practice when used for teaching nursing competencies.<br>AEMRs in skills and simulation settings replicates clinical environment and heightens realism.	Professional development.
Raghunathan et al. (2023)<br>*Factors in integrating academic electronic*	Australia	EMRs are vital, centralised, readily accessible clinical information sources which, not only facilitate better coordination of services and workflow	Fragmented application of EMRs in undergraduate nursing curricula.<br>Costs.	Ease of use.<br>Vendor and faculty support.	Self-directed learning modules for faculty.

medical records in nursing curricula: A qualitative multiple case studies approach<br>[47]		efficiencies, but improve safety, quality and timely care leading to better health<br>• Data management<br>• Development of digital literacy<br>• Practice using technologies<br>• Safe, quality patient care<br>• Understanding importance of data	Lack of faculty engagement.<br>Some students requiring additional preparation to use ICT.<br>Lack of sufficient equipment.<br>Lack of interoperability.<br>Full curriculum.	Timely product support when using commercial software.<br>Easy implementation.<br>AEMR championing and expertise around educational technologies.<br>Prior exposure to clinical EMRs among students and faculty.<br>Readily available teaching and learning content to be adapted into curriculum.<br>Provides examples of EMR application across three sites.	
Rees et al. (2025)<br>*Shaping the Future of Digital Health Education in Canada: Prioritizing Competencies for Health Care Professionals Using the Quintuple Aim*<br>[48]	Canada	Integration of informatics into education is essential for workforce capability and to enhance patient outcomes.<br>• Accessible care<br>• Data management<br>• Development of critical thinking and clinical reasoning<br>• Development of digital literacy<br>• Ethical use of technologies<br>• Safe, quality patient care<br>• Practice with technologies<br>• Understanding importance of data<br>• Workplace readiness of graduates	Nursing informatics instruction was relegated to theoretical definitions, concepts, and ideas scattered throughout various courses.<br>Burden of teaching bedside informatics skills left to clinical setting.	Professional practice strategies outlined by the Institute for Healthcare Improvement.<br>Experiential course activities applying theoretical and practical elements of nursing informatics.<br>Use of clinical practice councils with auditing of nursing charts.<br>Scaffolded modules.	Not described.
Repsha et al. (2020)<br>*Use of a Simulated Electronic Health Record to Support Nursing Student Informatics Knowledge and Skills*<br>[49]	USA	Preparation of graduates using informatics and technology is a crucial requirement in the transition to a competent, workforce ready RN.<br>• Development of digital literacy<br>• Practice using technologies	Nursing education programs are not uniform in the integration of NI into the curriculum.<br>Limited formal EHR training in curriculum.	Pre-intervention use of SANICS.<br>4 hours of high-fidelity simulation weekly.<br>Three of the simulation hours are completed in the simulation laboratory, while 1 hour is provided for preparation.<br>Simulated patient cases involve complex, acute situations presented as one or two patient assignments.<br>Post-intervention of SANICS.	Not described.
Risling (2017)<br>*Educating the nurses of 2025: Technology trends of the next decade*<br>[50]	Canada	Educators must focus on prepare the practitioners of the future.<br>• Data management<br>• Decision support<br>• Ethical use of technologies<br>• Evidence based practice<br>• Interdisciplinary communication<br>• Patient self-monitoring/ care	Integration of EHRs remain a challenge due to technical issues such as access speeds, content lag and interruptions related to malfunction.<br>A lack of consensus and specificity in managing digital best practice content in a digitised healthcare environment creates ongoing curricular challenges.	Nursing curriculums should include detailed digital best practice content covering legal and ethical concerns.<br>Comprehensive increase to informatics competencies throughout nursing curricular content supported by well-developed frameworks such as those	Educators should engage in future-casting about the potential evolution of nursing and not be limited by current practice paradigms.

		• Safe, quality patient care	Challenges with EHRs include underutilisation, language standardisation and lack of flexibility in design.<br>Resistance from nurses.	provided by TIGER or CASN (Canadian Association of Schools of Nursing).	
Royal College of Nursing (2018)<br>*Every Nurse an E-nurse Insights from a consultation on the digital future of nursing*<br>[51]	UK	Healthcare must change and modernise with a focus on technology and data.<br>• Cost effectiveness<br>• Data management<br>• Decision support<br>• Evidence based practice<br>• Interdisciplinary communication<br>• Population healthcare<br>• Rapid assessment<br>• Resource management<br>• Safe, quality patient care	Inadequacy of IT systems.<br>Lack of centralised procurement.<br>Chronic understaffing in clinical areas.<br>Lack of confidence about nursing and midwifery staff regarding digital competencies.<br>A degree of negativity about impact of digital technologies.<br>Lack of digital skills amongst nurses and midwives.	Provides a number of case study examples.	Not described.
Sapci & Sapci (2017)<br>*The effectiveness of hands-on health informatics skills exercises in the multidisciplinary smart home healthcare and health informatics training laboratories*<br>[52]	USA	Hands-on practice provides the opportunity for experiential learning and focuses on real-life challenges.<br>• Accessible care<br>• Authentic, real-world experience<br>• Data management<br>• Population healthcare<br>• Practice using technologies<br>• Safe, quality patient care	Little focus on innovation, remote patient monitoring education and experiential training.	Hands-on practice provides the opportunity for experiential learning.<br>Focuses on real life challenges.	More skilled laboratory staff to provide support during class.
Sorensen & Campbell (2016)<br>*Curricular path to value: Integrating an academic electronic health record*<br>[53]	USA	AEHR can provide students with learning opportunities through the navigation of technology, patient assessment, and decision making and expose them to standardised nursing language, evidence-based practice and ability to document patient information.<br>• Cost effectiveness<br>• Development of critical thinking and clinical reasoning<br>• Practice using technologies<br>• Recognition of importance of NI<br>• Reduction in clinical errors<br>• Safe, quality care<br>• Understanding importance of data	Nursing schools struggle to integrate AEHRs into their curricula, with shortage of competent faculty identified as barrier.<br>Majority of graduates do not feel prepared to access and use EHR in patient care.<br>Instructors' lack of time.<br>Resistance to new technology.	Integration of an AEHR with case studies better prepares nursing students to recognise critical patient cues within patient data, leading to more appropriate nursing diagnoses.	Faculty to share their innovative teaching strategies for AEHR integration.<br>Super-users/ faculty champions.<br>Skills and simulation laboratory coordinator provided further assistance with AEHR integration in scheduled laboratories.

Stunden et al. (2024)<br>*Nursing students' preparedness for the digitalised clinical environment in Australia: An integrative review*<br>[54]	Australia	Due to the fast-paced evolving world of digital technology all nurses need to be equipped with appropriate digital literacy and information communication technology (ICT) skills to provide efficient and safe nursing care.<br>• Data management<br>• Development of digital literacy<br>• Safe, quality patient care<br>• Understanding importance of data	Lack of EHRs with use of paper-based formats in academic settings.<br>Lack of nursing informatics education.<br>Many students in Australia lacked the digital literacy/competency skills required for university which would have a direct impact on their preparedness for the digitalised clinical setting.<br>Lack of faculty knowledge.	Integration of professional digital literacy competency standard framework.<br>To mirror the sophistication of health technology and informatics in the clinical setting, construction of a new curriculum is necessary for future graduate nurses to access health technology in Australia.<br>Integration of NI education and training competencies to be embedded into nursing curricula.<br>Integration of digital literacy courses.<br>Formalised training with use of EHRs.	It is imperative that academics are upskilled and trained in educating nursing students and equipping them with the required knowledge and digital literacy skills to function in the digitalised clinical setting.
Subramanian & Kleib (2023)<br>*Leveraging Clinical Preceptorship to Enhance Nursing Students Readiness in Digital Health*<br>[55]	Canada	Nursing must adapt to advance rapidly changing technologies and digitalisation.<br>• Accessible care<br>• Authentic, real-world experience<br>• Data management<br>• Development of digital literacy<br>• Ethical use of technologies<br>• Safe, quality patient care<br>• Understanding of importance of data	Disconnect between academic and clinical settings.<br>Nursing students are often described as tech-savvy; however, they face challenges in applying digital health technologies to patient care.<br>Lack of hands-on exposure to digital health technologies.<br>Limited access to digital health technologies on placement.	Mentorship in digital health leadership roles.<br>Opportunities to shadow nurses in NI roles.<br>Clinical placements with specialised digital health services and technologies.	Incentivised education programs for preceptors and clinical instructors.
Theron et al. (2019)<br>*Beyond Checklists: A Nursing Informatics Education Strategy for Undergraduate Nursing Students Appraising Health Information on Social Networking Sites (SNS)*<br>[56]	Canada	Growing expectation that students will be knowledgeable and able to integrate research and evidence-based sources into their practice.<br>• Ethical use of technologies<br>• Evidence based practice	Students' eHealth literacy skills are largely substandard.<br>Discrepancy between students' confidence in their ability to search and appraise health information and the quality of information they retrieved.	Development of the Digital health Assignment.	Not described.
Theron et al. (2017)<br>*Developing Digital Literacies in Undergraduate Nursing Studies: From Research to the Classroom*<br>[57]	Canada	Nurses use information and knowledge to inform practice and educate individuals, families and communities with information that will assist them in making healthcare decisions that will positively impact their quality of life.<br>• Ethical use of technologies<br>• Evidence based practice	Few student nurses learn about EHR in classroom context due to the lack of availability.<br>Lack of qualified faculty that understand technology from a conceptual, technical, and practice-based point of view.<br>Inaccuracy of online sources of information.<br>Many students lack skills to seek and evaluate online health information.	Perceptions of Students Regarding Their Digital Literacy Appraisal Skills.	Collaboration between educators and librarians provides students with resources to find and evaluate online health information.

Theron et al. (2017)<br>*Nursing Students' Perceived Learning from a Digital Health Assignment as Part of the Nursing Care for the Childbearing Family Course*<br>[58]	Canada	There is a need for building competency in the appraisal of online health information.<br>• Evidence based practice	Many students lack skills to appropriately find and evaluate online health information.<br>Students make quick and superficial judgments about the quality of online health information.<br>Students' actual abilities were lower than perceived abilities.	Students joined a closed Facebook group linked to the professor's course page.<br>Students selected an online site of interest and appraised site for trustworthiness using the TOHI (Trust in Online Health Information) Scale.<br>Students posted peer feedback to Facebook.	Educators can continue to work with librarians and faculty experts to increase students' knowledge and create opportunities for appraising not only online health information, but all health information.
Topol (2019)<br>*The Topol Review: Preparing the healthcare workforce to deliver the digital future. An independent report on behalf of the Secretary of State for Health and Social Care February 2019*<br>[59]	UK	Early, effective and sustained staff engagement at all levels, especially front-line staff, is a pre-requisite for technology enabled transformational change to be successful.<br>• Safe, quality patient care<br>• Data management	Uneven data quality.<br>Gaps in information governance.<br>Lack of expertise.<br>Resistance to change and scepticism.	Ensure genomics, data analytics and AI are prominent in undergraduate curricula.<br>Future healthcare professionals need to understand the possibilities of digital healthcare technologies and ethical practice and patient safety considerations.<br>Students must gain appropriate level of digital literacy at the outset of their study for their prospective career pathway.<br>Offer opportunities for healthcare students to work in areas such as engineering or computer science and equally attract graduates in these areas to begin a career in health, to create and implement technological solutions that improve care and productivity.	Not described.
Vottero (2017)<br>*Teaching Informatics to Prelicensure, RN-to-BSN, and Graduate Level Students*<br>[60]	USA	The current application of NI into curriculum provides opportunities for the use of unique designs and instructional techniques.<br>• Data management<br>• Decision supports<br>• Evidence based practice<br>• Safe, quality patient care	Many faculty equated NI with providing distance learning, online learning and web-based instruction.<br>Variations across curricula and programs.<br>Curriculum compression is experienced by many faculty who are trying to fit additional content requirements into an already tightly packed nursing curriculum.<br>Faculty's lack of knowledge or specialty certification in NI.	Provides a description of standalone course content or an integrated curriculum.	Not described.
Wilbanks et al. (2018)<br>*Electronic Health Records in Simulation*	USA	A major advantage of educational EHRs is the ability to teach students patient-centred care and disease state management using technology in a safe environment.<br>• Data management	Cost in acquiring the technology and software and human labour.<br>Requirement for internet connectivity.<br>Requirement for ICT staff.<br>Educational EHRs also require a lot of time from faculty who use it because they	Provides a list of best practices for AEHR implementation.	Faculty support, training, and adequate financial support are essential to a successful educational EHR.

Education: Literature Review and Synthesis<br>[61]		• Development of critical thinking and clinical reasoning<br>• Practice using technologies<br>• Recognition of importance of NI<br>• Safe, quality patient care	have to generate the clinical simulations and incorporate them into their curriculum.<br>Many faculty will often skip the documentation portion of the simulation if they are behind schedule and need to make room for other activities.		
Zhao et al. (2024)<br>*National survey on understanding nursing academics' perspectives on digital health education*<br>[62]	Australia	As nursing education and practice continue to evolve in the digital transformation of health, the digital health concepts in nursing education will continue to grow.<br>• Data management<br>• Development of digital literacy<br>• Ethical use of technologies<br>• Practice with technologies	Paucity of knowledge and expertise amongst nursing academics.<br>Insufficient digital literacy amongst nursing educators.<br>Costs of technologies.<br>Lack of awareness of digital health technologies.<br>Lack of integration of digital health technologies into undergraduate nursing curricula.<br>It can be challenging for nursing academics to determine the appropriate digital health skills within the range of clinical context in which the skills are required.<br>Lack of equity in some states and territories in Australia with access to digital health.	Ongoing engagement with technology stakeholders and experts.<br>Interdisciplinary collaboration.<br>Primary focus on role of nursing academics.<br>Development of resources and research networks.	Need to upskill nursing academics in the knowledge of digital health.<br>Collaboration between academics to upskill.<br>Capacity building required.<br>Professional development.

References

1. Almond, H.; Brice, S. Health Informatics Education Case Study: A Graduate Nurse Perspective. In Proceedings of the Studies in Health Technology and Informatics, 2025; pp. 2062-2063.
2. Almond, H.; Brice, S. Health Informatics Education Case Study: A Nurse Educator Perspective. In Proceedings of the Studies in Health Technology and Informatics, 2025; pp. 2064-2065.
3. Angel, V.M.; Friedman, M.H.; Friedman, A.L. Integrating Bar-Code Medication Administration Competencies in the Curriculum: Implications for Nursing Education and Interprofessional Collaboration. *Nursing Education Perspectives* **2016**, *37*, 239-241.https://doi.org/10.1097/01.NEP.0000000000000038
4. Baxter, P.M.; Andrew, L.A. Successful Integration of an Academic Electronic Health Record Into the Curriculum of an Associate Degree Nursing Program. *Nursing Education Perspectives* **2018**, *39*, 250-252.https://doi.org/10.1097/01.NEP.0000000000000255
5. Bonnel, W.; Vogel Smith, K.; Hober, C. *Teaching with Technologies in Nursing and the Health Professions: Strategies for Engagement, Quality, and Safety*; Springer: New York, 2018.
6. Booth, R.G.; Sinclair, B.; Brennan, L.; Strudwick, G. Developing and implementing a simulated electronic medication administration record for undergraduate nursing education: Using socio technical systems theory to inform practice and curricula. *CIN: Computers, Informatics, Nursing* **2017**, *35*, 131-139.https://doi.org/10.1097/CIN.0000000000000309
7. Booth, R.G.; Sinclair, B.; Strudwick, G.; Hall, J.; Tong, J.; Loggie, B.; Chan, R. Strategies Through Clinical Simulation to Support Nursing Students and Their Learning of Barcode Medication Administration (BCMA) and Electronic Medication Administration Record (eMAR) Technologies. In *Health Professionals' Education in the Age of Clinical Information Systems, Mobile Computing and Social Networks*, Shachak, A., Borycki, E.M., Reis, S.P., Eds.; Academic: Cambridge, 2017; pp. 246-266.
8. Bove, L.A.; Sauer, P. Nursing Faculty Informatics Competencies. *CIN: Computers, Informatics, Nursing* **2023**, *41*, 18-23.https://doi.org/10.1097/CIN.0000000000000894
9. Brunel, M.; Allert, J. Integrating Experiential Informatics and Quality Improvement in Baccalaureate Education. *Nursing Education Perspectives* **2025**, *46*, 124-125.https://doi.org/10.1097/01.NEP.0000000000001236
10. Brunner, M.; McGregor, D.; Keep, M.; Janssen, A.; Spallek, H.; Quinn, D.; Jones, A.; Tseris, E.; Yeung, W.; Togher, L.; et al. An eHealth capabilities framework for graduates and health professionals: Mixed-methods study. *Journal of Medical Internet Research* **2018**, *20*, 1-9.https://doi.org/10.2196/10229
11. Burke, M.S.; Ellis, D.M. Electronic Health Records: Describing Technological Stressors of Nurse Educators. *Nurse Educator* **2016**, *41*, 46-48.https://doi.org/10.1097/NNE.0000000000000196

12. Chauvette, A.; Kleib, M.; Paul, P. Developing nursing students' informatics competencies - A Canadian faculty perspective. *International Journal of Nursing Education Scholarship* **2022**, *19*, 1-11.https://doi.org/10.1515/ijnes-2021-0165
13. Chauvette, A.; Paul, P.; Kleib, M. The impetus of COVID-19 in transforming nursing education through informatics. *Quality advancement in nursing education* **2022**, *8*, 1-15.https://doi.org/10.17483/2368-6669.1355
14. Clancy, T.R. National Nursing Informatics Deep Dive Program Integrating AACN Essentials for Information Management and Patient Care Technologies Across the Continuum. **2015**.
15. Clever Together. *A vision for the Changing Faculty Role: Preparing Students for the Technological World of Health Care*; National League for Nursing (NLN): 2015.
16. Cross, L.; Kendrick, A. Artificial intelligence and the meta-paradigm. *Teaching and Learning in Nursing* **2025**.https://doi.org/10.1016/j.teln.2025.08.015
17. Cummings, E.; Borycki, E.M.; Madsen, I. Teaching Nursing Informatics in Australia, Canada and Denmark. In *Context Sensitive Health Informatics: Many Places, Many Users, Many Contexts, Many Uses*, Borycki, E.M., Kushniruk, A.W., Kuziemsky, C.E., Nohr, N., Eds.; Studies in Health Technology and Informatics; IOS: 2015; Volume 218, pp. 39-44.
18. Cummings, E.; Whetton, S.; Mather, C. Integrating Health Informatics Into Australian Higher Education Health Profession Curricula. In *Health Professionals' Education in the Age of Clinical Information Systems, Mobile Computing and Social Networks*, Shachak, A., Borycki, E.M., Reis, S.P., Eds.; Academic: Cambridge, 2017; pp. 323-343.
19. Doerner, M.E.; Connerton, C.S.; Wijesuriya, U. Student growth in informatics competencies after a nursing informatics course. *Teaching and learning in nursing* **2025**, *20*, e1240-e1244.https://doi.org/10.1016/j.teln.2025.07.012
20. Forman, T.M.; Armor, D.A.; Miller, A.S. A Review of Clinical Informatics Competencies in Nursing to Inform Best Practices in Education and Nurse Faculty Development. *Nursing Education Perspectives* **2020**, *41*, 1-7.https://doi.org/10.1097/01.NEP.0000000000000588
21. Forman, T.M.; Flores, D.; Miller, A.S. An Integrative Literature Review of the Use of Electronic Health Records for Clinical Nursing Education. *Journal of Informatics Nursing* **2020**, *5*, 28-44
22. Foster, M.; Sethares, K. Current Strategies to Implement Informatics into the Nursing Curriculum: An Integrative Review. *Online Journal of Nursing Informatics* **2017**, *21*, 4-1
23. Gambo, J.M.; Bahreman, N.T.; Watties-Daniels, D.; Neal, M.; Swoboda, S.M. Can Mobile Technology Enhance Learning and Change Educational Practice? *CIN: Computers, Informatics, Nursing* **2017**, *35*, 375-380.https://doi.org/10.1097/CIN.0000000000000380
24. Hamilton, H.; Iradukunda, F.; Aselton, P. The Integration of Telehealth in Nursing Education: A New Frontier. *Journal of Informatics Nursing* **2021**, *6*, 18-25
25. Harerimana, A.; Wicking, K.; Biedermann, N.; Yates, K. Nursing informatics in undergraduate nursing education in Australia before COVID-19: A scoping review. *Collegian* **2022**, 527-539.https://doi.org/10.1016/j.colegn.2021.11.004
26. Hay, B.; Carr, P.J.; Dawe, L.; Clark-Burg, K. “iM Ready to Learn”: Undergraduate Nursing Students Knowledge, Preferences, and Practice of Mobile Technology and Social Media. *CIN: Computers, Informatics, Nursing* **2017**, *35*,
27. Hern, M.J.; Key, M.; Goss, L.K.; Owens, H. Facilitating adoption of informatics and meaningful use of electronic health records with nursing faculty. *Journal of Nursing Education and Practice* **2015**, *5*, 118-126.https://doi.org/10.5430/jnep.v5n3p118
28. Honey, M.; Collins, E.; Britnell, S. Identifying How to Support Nurse Educators Nationally to Teach Nursing Informatics. In *Nurses and Midwives in the Digital Age*, Honey, M., Ronquillo, C., Lee, T., Westbrooke, L., Eds.; Stud Health Technol Inform; IOS: 2021; Volume 284, pp. 124-129.
29. Hovenga, E.J.S.; Grain, H. Learning, Training and Teaching of Health Informatics and its Evidence for Informaticians and Clinical Practice. In *Evidence-Based Health Informatics*, Ammenwerth, E., Rigby, M., Eds.; Studies in Health Technology and Informatics; IOS: 2016; Volume 222, pp. 336-354.
30. Kleib, M.; Arnaert, A.; Nagle, L.; Sugars, R. Education About Digital Health and Artificial Intelligence and Learning Needs: Perspectives of Undergraduate Nursing Students. *Quality Advancement in Nursing Education* **2025**, *11*.https://doi.org/10.17483/ctzr1e66
31. Kleib, M.; Arnaert, A.; Nagle, L.M.; Ali, S.; Idrees, S.; Costa, D.d.; Kennedy, M.; Darko, E.M. Digital Health Education and Training for Undergraduate and Graduate Nursing Students: Scoping Review. *JMIR Nursing* **2024**, *7*, 1-17.https://doi.org/10.2196/58170
32. Kleib, M.; Olson, K. Evaluation of an informatics educational intervention to enhance informatics competence among baccalaureate nursing students. *Knowledge Management & E-Learning* **2015**, *7*, 395-411.https://doi.org/10.34105/j.kmel.2015.07.026
33. Lam, M.; Hines, M.; Lowe, R.; Nagarajan, S.; Keep, M.; Penman, M.; Power, E. Preparedness for eHealth: Health Sciences Students’ Knowledge, Skills, and Confidence. *Journal of Information Technology Education: Research* **2016**, *15*, 305-334.https://doi.org/10.28945/3523
34. Lokmic-Tomkins, Z.; Gray, K.; Cheshire, L.; Parolini, A.; Sharp, M.; Tarrant, B.; Hill, N.; Rose, D.; Webster, M.; Virtue, D.; et al. Integrating interprofessional electronic medical record teaching in preregistration healthcare degrees: A case study. *International journal of medical informatics (Shannon, Ireland)* **2023**, *169*, 104910.https://doi.org/10.1016/j.ijmedinf.2022.104910
35. Luo, S.; Kalman, M. A technology training protocol for meeting QSEN goals: Focusing on meaningful learning. *Nursing Forum* **2018**, *53*, 20-26.https://doi.org/10.1111/nuf.12214
36. Mather, C.; Cummings, E. Empowering learners: Using a triad model to promote eHealth literacy and transform learning at point of care. *Knowledge management & e-learning* **2015**, *7*, 629-645.https://doi.org/10.34105/j.kmel.2015.07.041
37. Mather, C.; Cummings, E. Issues for Deployment of Mobile Learning by Nurses in Australian Healthcare Setting. In *Nursing Informatics 2016*, Sermeus, W., Procter, P.M., Weber, P., Eds.; Studies in Health Technology and Informatics; IOS: 2016; pp. 277-281.
38. Mather, C.; Cummings, E.; Nichols, L. Social Media Training for Professional Identity Development in Undergraduate Nurses. In *Nursing Informatics 2016*, Sermeus, W., Procter, P.M., Weber, P., Eds.; IOS: Amsterdam, 2016; Volume 225, pp. 344-348.
39. McGregor, D.; Keep, M.; Brunner, M.; Janssen, A.; Quinn, D.; Avery, J.; Togher, L.; Shaw, T. Preparing E-Health Ready Graduates: A Qualitative Focus Group Study In *Integrating and Connecting Care*, Schaper, L.K., Whetton, S., Ryan, A., Eds.; IOS: 2017; Volume 239, pp. 91-96.
40. Mollart, L.; Stubbs, M.; Noble, D.; Koizumi, N.; Crowfoot, G. Student confidence and knowledge with electronic medical records through on-ward simulation: An evaluation study. *Clinical Simulation in Nursing* **2025**, *106*.https://doi.org/10.1016/j.ecns.2025.101792
41. O'Connor, S.; Andrews, T. Mobile Technology and Its Use in Clinical Nursing Education: A Literature Review. *Journal of Nursing Education* **2015**, *54*, 137-144.https://doi.org/10.3928/01484834-20150218-01
42. O'Connor, S.; Hubner, U.; Shaw, T.; Blake, R.; Ball, M. Time for TIGER to ROAR! Technology Informatics Guiding Education Reform. *Nurse Education Today* **2017**, *58*, 78-81.https://doi.org/10.1016/j.nedt.2017.07.014

43. O'Connor, S.; LaRue, E. Integrating informatics into undergraduate nursing education: A case study using a spiral learning approach. *Nurse Education in Practice* **2021**, *50*, 1-6.https://doi.org/10.1016/j.nepr.2020.102934
44. Pobocik, T. Using an Educational Electronic Documentation System to Help Nursing Students Accurately Identify Patient Data. *International Journal of Nursing Knowledge* **2015**, 26-34.https://doi.org/10.1111/2047-3095.12032
45. Raghunathan, K.; McKenna, L.; Peddle, M. Use of academic electronic medical records in nurse education: A scoping review. *Nurse Education Today* **2021**, *101*, 1-16.https://doi.org/10.1016/j.nedt.2021.104889
46. Raghunathan, K.; McKenna, L.; Peddle, M. Utilisation of academic electronic medical records in pre-registration nurse education: A descriptive study. *Collegian* **2022**, *29*, 645-653.https://doi.org/10.1016/j.colegn.2022.03.005
47. Raghunathan, K.; McKenna, L.; Peddle, M. Factors in integrating academic electronic medical records in nursing curricula: A qualitative multiple case studies approach. *Nurse education today* **2023**, *120*, 105626.https://doi.org/10.1016/j.nedt.2022.105626
48. Rees, G.; Nowell, L.; Risling, T. Shaping the Future of Digital Health Education in Canada: Prioritizing Competencies for Health Care Professionals Using the Quintuple Aim. *JMIR Medical Education* **2025**, *11*.https://doi.org/10.2196/75904
49. Repsha, C.; Morse, B.; Lee, S.E.; Katz, J.; Burrows, E.; Teates, J. Use of a Simulated Electronic Health Record to Support Nursing Student Informatics Knowledge and Skills. *CIN: Computers, Informatics, Nursing* **2020**, *38*, 55-59.https://doi.org/https://dx.doi.org/10.1097/CIN.0000000000000618
50. Risling, T. Educating the nurses of 2025: Technology trends of the next decade. *Nurse Education in Practice* **2017**, *22*, 89-92.https://doi.org/10.1016/j.nepr.2016.12.007
51. Royal College of Nursing. Every Nurse an E-nurse Insights from a consultation on the digital future of nursing. **2018**.
52. Sapci, A.H.; Sapci, H.A. The effectiveness of hands-on health informatics skills exercises in the multidisciplinary smart home healthcare and health informatics training laboratories. *Applied Clinical Informatics* **2017**, *8*, 1184-1196.https://doi.org/10.4338/ACI-2017-08-RA-0136
53. Sorensen, J.; Campbell, L. Curricular path to value: Integrating an academic electronic health record. *Journal of Nursing Education* **2016**, *55*, 716-719.https://doi.org/10.3928/01484834-20161114-10
54. Stunden, A.; Ginige, A.; O'Reilly, R.; Sanagavarapu, P.; Heaton, L.; Jefferies, D. Nursing students' preparedness for the digitalised clinical environment in Australia: An integrative review. *Nurse Education in Practice* **2024**, *75*.https://doi.org/10.1016/j.nepr.2024.103908
55. Subramanian, S.; Kleib, M. Leveraging Clinical Preceptorship to Enhance Nursing Students' Readiness in Digital Health. *Quality Advancement in Nursing Education* **2023**, *9*.https://doi.org/10.17483/2368-6669.1412
56. Theron, M.J.; Astle, B.; Dixon, D.; Redmond, A. Beyond Checklists: A Nursing Informatics Education Strategy for Undergraduate Nursing Students Appraising Health Information on Social Networking Sites (SNS). *Quality Advancement in Nursing Education* **2019**, *5*, 1-18.https://doi.org/https://doi.org/10.17483/2368-6669.1174
57. Theron, M.; Borycki, E.M.; Redmond, A. Developing Digital Literacies in Undergraduate Nursing Studies: From Research to the Classroom. In *Health Professionals' Education in the Age of Clinical Information Systems, Mobile Computing and Social Networks*, Shachak, A., Reis, S.P., Borycki, E.M., Eds.; Elsevier: 2017; pp. 149-173.
58. Theron, M.; Redmond, A.; Borycki, E.M. Nursing Students' Perceived Learning from a Digital Health Assignment as Part of the Nursing Care for the Childbearing Family Course. In *Building Capacity for Health Informatics in the Future*, Lau, F., Bartle-Clar, J.A., Bliss, G., Eds.; Studies in Health Technology and Informatics; IOS: 2017; Volume 234, pp. 328-335.
59. Topol, E. *The Topol Review: Preparing the healthcare workforce to deliver the digital future. An independent report on behalf of the Secretary of State for Health and Social Care February 2019.*; NHS (National Service), 2019.
60. Vottero, B.A. Teaching Informatics to Prelicensure, RN-to-BSN, and Graduate Level Students. *Nurse Educator* **2017**, *42*, 22-26.https://doi.org/10.1097/NNE.0000000000000414
61. Wilbanks, B.A.; Watts, P.I.; Epps, C.A. Electronic Health Records in Simulation Education: Literature Review and Synthesis. *Simulation in Healthcare* **2018**, *13*, 261-267.https://doi.org/10.1097/SIH.0000000000000288
62. Zhao, L.; Abdolkhani, R.; Walter, R.; Petersen, S.; Butler-Henderson, K.; Livesay, K. National survey on understanding nursing academics' perspectives on digital health education. *Journal of advanced nursing* **2024**, *80*, 4888-4899.https://doi.org/10.1111/jan.16163
